# Pathways linking greenspace to behavioural problems in Polish children

**DOI:** 10.1016/j.heliyon.2024.e31435

**Published:** 2024-05-18

**Authors:** Nitika Singh, Dorota Buczyłowska, Clemens Baumbach, Jakub Bratkowski, Yarema Mysak, Maja Wierzba-Łukaszyk, Katarzyna Sitnik-Warchulska, Krzysztof Skotak, Małgorzata Lipowska, Bernadetta Izydorczyk, Marcin Szwed, Angel M. Dzhambov, Iana Markevych

**Affiliations:** a Institute of Psychology, Jagiellonian University, Kraków, Poland; b Doctoral School of Social Sciences, Jagiellonian University, Kraków, Poland; c Institute and Clinic for Occupational, Social and Environmental Medicine, University Hospital, Ludwig-Maximilians-University, Munich, Germany; d Institute of Environmental Protection-National Research Institute, Warsaw, Poland; e Faculty of Management and Social Communication, Institute of Applied Psychology, Jagiellonian University, Kraków, Poland; f Institute of Psychology, University of Gdańsk, Gdańsk, Poland; g Department of Hygiene, Faculty of Public Health, Medical University of Plovdiv, Plovdiv, Bulgaria; h Institute for Highway Engineering and Transport Planning, Graz University of Technology, Graz, Austria; i Research Group “Health and Quality of Life in a Green and Sustainable Environment”, SRIPD, Medical University of Plovdiv, Plovdiv, Bulgaria; j Environmental Health Division, Research Institute at Medical University of Plovdiv, Medical University of Plovdiv, Plovdiv, Bulgaria

**Keywords:** Green space, Behavioural problems, Children, Mental health, Neighbourhood cohesion, Physical activity

## Abstract

**Background:**

Previous cross-sectional studies have found a beneficial relationship between greenspace and children's behaviour. Nevertheless, evidence on the mechanisms underlying this association remains scant. We examined whether the availability of greenspace was related to fewer behavioural problems in Polish children and investigated potential mechanisms.

**Methods:**

Data were obtained from the case-control NeuroSmog study, in which children with and without attention deficit hyperactivity disorder (ADHD) were tested from October 2020 to September 2022. The analytic sample comprised 679 children aged 10–13 years. Parents reported internalizing, externalizing, and total behavioural problems using the Child Behaviour Check List (CBCL), as well as information about the presence of a domestic garden and potential mediators: greenspace perception, neighbourhood social cohesion, and physical activity. Tree and grass covers were extracted in 500 m and 1 km buffers around lifelong residences. Structural equation modelling (SEM) was used to examine the psychosocial pathways linking the greenspace metrics to behavioural problems.

**Results:**

Greenspace was only indirectly related to fewer behavioural problems. Specifically, tree cover was related to greater levels of physical activity which, in turn, was related to fewer internalizing and total behavioural problems. Tree cover and presence of garden were related to greenspace perception which, in turn, was associated with higher neighbourhood social cohesion which, in turn, was linked to fewer behavioural problems. The patterns of associations in children without ADHD were very similar to those in the full sample except that the associations from garden to greenspace perception and from physical activity to total behavioural problems were no longer significant. The only association persisted among girls was from neighbourhood social cohesion to behavioural problems and among boys were from tree cover to physical activity and tree cover and garden to greenspace perception.

**Conclusion:**

Trees and garden, but not grass, are linked to fewer behavioural problems through greenspace perception, neighbourhood social cohesion, and physical activity in Polish children.

## Highlights

• Greenspace already linked to fewer behavioural problems but mechanisms unknown.
• We used data of 679 Polish 10- to 13-year-old children with and without ADHD.
• Greenspace perception, neighbourhood social cohesion, and physical activity tested as mediators.
• Pathway 1: tree cover .→ physical activity → fewer total and internalizing problems
• Pathway 2: tree cover/garden .→ greenspace perception → neighbourhood cohesion → fewer problems

## 1 Introduction

Behavioural problems in childhood are a matter of concern, since they are common and constitute the basis for the development of mental disorders of the same kind in adulthood, for instance, depression, anxiety, and disruptive and substance use disorders [1,[2], [3]]. Internalizing behavioural problems such as anxiety, depression, and withdrawal, as well as externalizing behavioural problems such as breaking social rules and aggression, are among the most common mental disorders (Achenbach TM, Rescorla LA, 2007 [4], [5]]). Worldwide, one in seven 10- to 19-year-olds suffers from mental disorders, accounting for 13 % of the global burden of disease [6].

According to two prominent environmental psychology theories, attention restoration theory (ART) [7,8] and stress reduction theory (SRT) [9], natural environments, such as greenspace, can have positive effects on cognitive and emotional states. ART posits that exposure to natural environments can restore cognitive function by providing a break from everyday stimuli and promoting profound sense of wonder, engagement, and awe when immersed in nature, thereby fostering fascination. Moreover, natural environments often offer rich sensory experiences that stimulate curiosity and exploration, fostering creativity and problem-solving skills. SRT suggests that exposure to natural environments can reduce physiological and psychological stress and promote relaxation. These theories have important implications for child mental health and well-being, as well as cognitive and emotional development.

Growing evidence suggests that nature exposure is beneficial for mental and physical health, especially for longevity, cardiometabolic health, birth weight, physical activity, quality of sleep, and mental health [10]. Exposure to nature may reduce stress [11,[9], [12]] and rumination [13,14], improve symptoms of depression [[15], [16]], sleep quality [[17], [18]], inattention [7,8,19], and cognitive functioning [20]. Viewing nature make us happier [21], more trusting and generous [22], and nature sounds have a restorative stress-relieving effect [23]. Greenspace has been shown to have a profound impact on both children and adults, yet it affects each group in different ways. Adults often use nature as a means of finding peace and solace in their busy lives and can benefit from the calming and rejuvenating effects of spending time outdoors (Barton and Rogerson 2017 [10]). Children living in greener environments have, in particular, opportunities to engage in outdoor play or other more structured forms of physical activity; they can also connect with, explore, and learn about their environment, and develop a sense of belonging to the natural world [24,25]. Research has shown that children who grow up in a house with a garden or more greenspace around their residence tend to be more physically active [26], perceive nature positively [27], and develop a sense of social cohesion [28]. Previous research suggests that higher levels of physical activity, greenspace perception, and social cohesion are related to fewer behavioural problems in children [29,30]. Tree cover may be of particular important as Pearson and colleagues [31] found that higher levels of visible nature from classroom windows were associated with lower externalizing behaviour problem scores and the relationship was consistent for visible trees, but not other nature types.

Although prior studies have explored the relationship between exposure to nature and behavioural problems in children and reported beneficial associations (Zare Sakhvidi et al., 2022), no studies, to our knowledge, have specifically investigated the complex underpinning mechanistic links, except for one recent study by Dzhambov et al. [32] where the authors scrutinized a complex interplay of sleep problems, passive smoking, body mass index, and related biomarkers that can be on the pathway to behavioural problems, and uncovered that passive smoking was a driving mechanism. However, no study has looked whether nature affects behavioural problems in children with attention deficit hyperactivity disorder (ADHD), a potentially vulnerable group that may benefit from exposure to greenspace [33]. ADHD, characterized by inattention, impulsivity, and hyperactivity, is of global public health concern, affecting 5 %–8 % of children [34]. Nature provides a calm atmosphere, diverse sensory stimulation and opportunities for free movement that aligns well with the needs of children with ADHD, promoting their overall well-being and development. It stands to reason that mechanisms supporting normal neurodevelopment become activated to a different extent and potentially hold different valence for children with and without ADHD.

To expand our understanding of this subject matter, our study investigated whether greater greenspace availability was associated with fewer behavioural problems in Polish children aged 10–13 years allowing for psychosocial pathways through perceived greenspace, neighbourhood social cohesion, and physical activity. In addition, we checked whether this association differed in children with ADHD compared to their neurotypically developing peers.

## 2 Methods

### 2.1 Study population

The current secondary analysis utilizes data collected in the case-control study NeuroSmog, originally designed to assess long-term effects of air pollution on the developing brain of children with and without ADHD. Data collection was carried out between October 2020 and September 2022 in 18 towns in southern Poland. Cases were recruited by assessing psychologists and referred to from mental health hospitals and school psychologists, while population-based controls were randomly sampled from randomly selected schools. ADHD was diagnosed based on comprehensive testing by clinical psychologists, and later, verified by three senior clinical psychologists, in accordance with the International Classification of Diseases 11th Revision (ICD-11) criteria [34]. Children had to be aged 10–13 years, fluent Polish speakers, attend school in one of the selected towns, study in classes IV, V, or VI, and have at least average intelligence. Children born before 35 weeks, with birth weight less than 2500 g, diagnosed with serious comorbidities, including mental and neurological disorders, with contraindications to magnetic resonance imaging (MRI), and children in the ADHD sample that did not meet the ADHD criteria of ICD-11 were excluded. All children underwent comprehensive psychological evaluation and MRI scanning. More details can be found elsewhere [35].

Initially, 741 participants were recruited and tested. However, 62 participants were excluded because exposure, mediator, or confounder data were not available. Our final sample size comprised 679 children (476 children without ADHD and 203 children with ADHD) (Fig. S1).

**Fig. 1:**
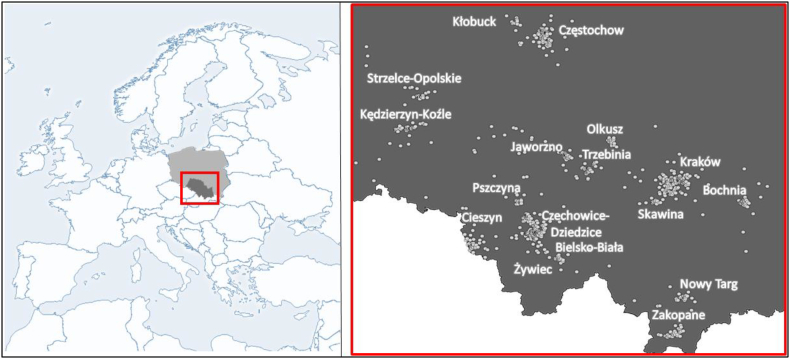
Study area in Europe (left pane) and lifelong residential addresses of the participants (white dots) across the study area (right pane). Note: Many children have resided at more than one address.

In line with its main goal, NeuroSmog's sample size was not based on power calculations but was maximized under the financial and logistic constraint that every child had to undergo MRI scanning. Fig. 1 shows lifelong residential addresses and study area locations for study participants throughout Poland.

NeuroSmog has been approved by the ethical committee of the Institute of Psychology, Jagiellonian University, Kraków, Poland (# KE_24042019A). Written informed consent was collected from the legal guardians of participants, and written informed assent was received from each participant.

### 2.2 Behavioural problems assessment

Behavioural problems were measured with the Polish version of the Child Behaviour Checklist (CBCL) for ages 6 to 18 [4,36,37]. CBCL is a component of the Achenbach System of Empirically Based Assessment (ASEBA) that is used to assess behavioural and emotional problems in children and adolescents over the last 6 months, and it was filled in by parents [4]. CBCL is a widely used tool with strong validity and reliability [[38], [39]]. It consists of 113 questions scored on a scale of 0–2 points (0 = not true, 1 = somewhat or sometimes true, 2 = very or often true). The questions cover eight ‘narrowband’ syndrome scales that form two ‘broadband’ scales (Fig. S2). Narrowband syndrome scales are aggressive behaviour, anxious/depressed, attention problems, rule-breaking behaviour, somatic complaints, social problems, thought problems, and withdrawn/depressed. Broadband scales are internalizing problems (sum of anxious/depressed, withdrawn/depressed, and somatic complaints scores) and externalizing problems (sum of rule-breaking and aggressive behaviour scores). The total problems score combines all problem scores in CBCL (Achenbach TM, Rescorla LA, 2007 [4]). In our analysis, internalizing, externalizing, and total problems were treated as outcomes. The internal consistency of the scales in our data was good to excellent (Cronbach's alpha was 0.89, 0.92, and 0.96, respectively).

### 2.3 Exposure to greenspace

Exposure to greenspace was represented by percentage of tree and grass/shrub cover (henceforth referred to as “grass”) in a 500 m buffer around home addresses, as well as presence of a home garden at the present address. Due to their widespread availability and potential relevance for children's well-being, these indicators are commonly used as proxies for greenspace exposure in studies exploring the relationship between greenspace and behavioural problems in children [40]. We treated grass and tree cover separately because we assumed associations with tree cover to be stronger [41,42]. The 500 m buffer was selected as studies on independent mobility of children of similar ages have shown that they report travelling no more than 500 m to access green space (Villanueva et al., 2012; Hand et al., 2018). Tree and grass cover in 1 km buffers were considered alternative exposures in a sensitivity analysis.

Lifetime addresses of children were collected using paper and pencil questionnaires (Markevych et al., 2022; Supplement B). Later, addresses were transferred from paper to a digital spreadsheet and geocoded using the R (R core Team, 2012) *geocode(.)* function of the package *ggmap* (Kahle and Wickham, 2013).

**Fig. 2:**
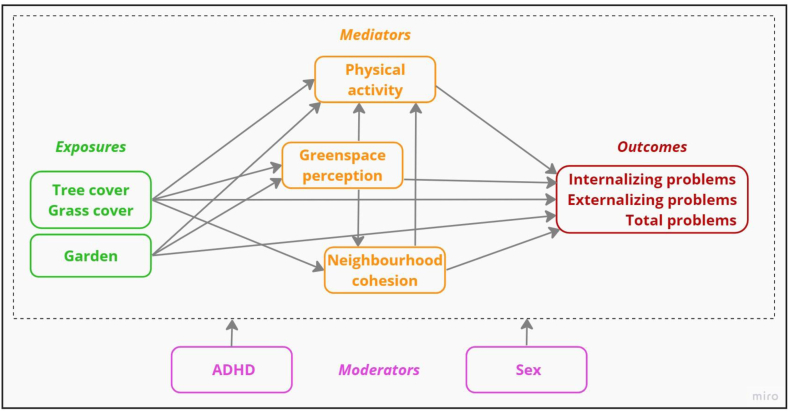
Conceptual diagram depicting theoretically indicated pathways linking greenspace to behavioural problems. Age, sex, parental education, perceived financial situation, and town size are confounders.

Information on tree and grass area were calculated in square meters from the Polish national land cover data collection BDOT10k (“baza danych obiektów topograficznych 10 k” = “database of topographic objects 10 k”) ([43]; Fig. 2). To sum up, first, gdal's rasterize function (GDAL version 3.0.4) (GDAL/OGR contributors, 2022) was used to convert tree and grass cover vector layers into rasters at a resolution of 20 m × 20 m. Second, cover in square meters within 500 m and 1 km Euclidean buffers were calculated for each of the relevant layers by focal sum in the geographic information system ArcGIS Pro 2.5.1 (ESRI, 2011). Assignments were conducted using the tifffile package version 2022.5.4 in Python version 3.9.6. Lifelong exposure to greenspace was calculated as the average exposure over all residential addresses weighted by duration of residence at each address. Afterwards, tree and grass variables were converted into percentages. Fig. S3 shows the greenspace layers for the study area.

Information on the presence of a domestic garden was based on the parental answer to the yes-no question: ‘Is there a garden present in your child's current home?’

### 2.4 Potential mediators

#### 2.4.1 Perception of greenspace

Instead of treating perceived greenspace as a stand-alone exposure, we considered it as a mediator, in line with previous studies (Dzhambov et al., 2018a, 2018b). Five items tapping different aspects of greenspace exposure were used to construct this greenspace perception variable: (1) vegetation degree of the neighbourhood, (2) green window view from home, (3) quality of greenspace, (4) time spent in neighbourhood greenspace, and (5) time spent in greenspace not in the immediate vicinity of the home (Markevych et al., 2022; Supplement A). Parents rated each item on a Likert scale with response options ranging from “Strongly disagree” (coded as 1) to “Strongly agree” (coded as 7) with higher scores indicating greater amounts of perceived greenspace. The mean score of the responses to the five items was used in the analysis. Internal consistency of the scale in our data was good (Cronbach's alpha = 0.78).

#### 2.4.2 Neighbourhood social cohesion

Perceived neighbourhood social cohesion (henceforth referred to as “neighbourhood cohesion”) was measured with the Brief Form of the Perceived Neighbourhood Social Cohesion Questionnaire (PNSC–BF) [44]. PNSC-BF is a nine-item questionnaire covering the following three dimensions: “Trust” (e.g. trust in people, including members of the neighbourhood who are not personally known), “Attachment to neighbourhood” (e.g. feeling part of the community), and “Tolerance and respect” (e.g. reciprocal tolerance among the community) (Markevych et al., 2022; Supplement A). Each item was rated by parents on a seven-point Likert scale. The neighbourhood cohesion score is the sum of responses and ranges from 9 to 63, with higher scores indicating higher cohesion. The score's Cronbach's alpha of 0.93 indicates excellent internal consistency in our data.

#### 2.4.3 Physical activity

Parents reported the frequency and duration of physical activity of their child by answering two questions adopted from the RHINESSA study: “How often (How many hours per week) does your child usually do strenuous physical activity outside of school that makes her or him get out of breath or sweat more than usual (e.g., playing team sports, dancing, swimming, etc.)?” [45,46]. Response options for physical activity frequency were: Every day, 4–6 times per week, 1–3 times per week, At least once per month, Less than once per month, Never, and Don't know. Physical activity duration had the following response options: More than 6 h per week, 5–6 h per week, 3–4 h per week, 1–2 h per week, Less than 1 h per week, None, and Don't know.

### 2.5 Confounders and moderators

We selected the following confounders: age (days expressed in years), sex (female vs male), socio-economic status (SES; measured by summarising parental education based on the lowest education of either parent; low vs medium vs high), and perceived financial situation in 2019 (it was very difficult/it was quite difficult/we just managed to make ends meet vs we were doing alright vs we were living comfortably), and town size (small vs large). ADHD diagnosis (Section 2.1) was considered a moderator.

### 2.6 Statistical analysis

The statistical software R (R Core Team, 2012), version 4.2.2, was used for data preparation and statistical analysis. For categorical variables, descriptive properties of the analytic sample are reported as frequencies and percentages and for numerical variables as arithmetic means, standard deviations, minimums, and maximums. Histograms were used to examine the distribution of the numerical variables and showed that internalizing, externalizing, and total problems were right-skewed while greenspace perception and social cohesion were left-skewed. Visual inspection of scatter plots revealed no outliers. Pairwise correlation coefficients were plotted to visually explore the strength and direction of univariate relationships between the variables in the dataset. Pearson correlation coefficients were used for pairs of numerical variables, including ordinal variables converted to numerical, phi coefficients were used for pairs of binary variables, and point-biserial correlation coefficients for numerical-binary variable pairs. We interpreted correlation coefficients with absolute values ranging from 0.3 to 0.7 as moderate and those above 0.7 as strong correlations [47].

We used structural equation modelling (SEM) to investigate hypothesized pathways from tree cover, grass cover, and garden at one end to behavioural problems at the other end. SEM allows testing for serial mediation and interplay of mediators in one and the same model rather than in a piecewise fashion (Dzhambov et al., 2020). We tested whether the associations of greenspace indicators with internalizing, externalizing, and total problems were collectively mediated by perceived greenspace, neighbourhood cohesion, and physical activity and moderated by ADHD and sex. (Fig. 2).

Before running SEM, numerical variables were transformed into z-scores because they were on different scales. Thus, their resulting effect estimates were standardized. Note that the term “effect” is not meant to imply causality. We are merely adhering to the standard terminology in the mediation analysis literature. Internalizing, externalizing, and total behavioural problems were allowed to have non-zero pairwise covariances. The indicators of physical activity were specified to load into a latent variable, and the same was done with the SES indicators. The model parameters were estimated by diagonally weighted least squares (DWLS) estimation (Muthen, 1993) and bootstrapping with 1000 draws was used to construct the standard errors and 95 % confidence intervals (CI) of all direct and indirect effects in the model. For a given variable in the SEM model, a direct effect corresponds to a path coefficient. An indirect effect is defined as a product of path coefficients. A total indirect effect is a sum of indirect effects. A total effect is the sum of direct effect and total indirect effect. In addition to the main SEM model, we ran separate SEM models for children with and without ADHD, since children with ADHD suffer from a higher level of externalizing and total behavioural problems and we treated ADHD as a moderator (Speyer et al., 2021). Additionally, we ran separate SEM models for girls and boys, since they use neighbourhood differently [48] and may benefit from different aspects of neighbourhood [49] and we treated sex as a moderator. We followed Hu and Bentler's (1999) recommendations to assess goodness of fit: a non-significant χ2 (p-value >0.05), a root mean square error of approximation (RMSEA) ≤ 0.06, a standardized root mean square residual (SRMR) ≤ 0.08, and a comparative fit index (CFI) ≥ 0.95. We also reported chi-square to degrees of freedom ratio (χ2/df) and Tucker-Lewis Index (TLI). Estimates whose 95 % bootstrap Cis do not contain zero are assumed to be significant. The SEM model was fitted with version 06–12 of R's lavaan package (Rosseel, 2012).

## 3 Results

### 3.1 Analytic sample descriptives

Table 1 shows the characteristics of the sample used for analysis. Excluded sample (N = 56) was not different from the analytic sample (Table S1). 203 (29.9 %) of the 679 participants had ADHD. 346 (51.0 %) children attended school in a large town. The mean age was 11.3 years and 58.8 % of participants were male. Girls made up only 25.1 % of the ADHD population, compared to 48.1 % of children without ADHD. Parents of children with ADHD were more likely to have attained low education compared to parents of children without ADHD. Children with ADHD tended to live in large towns and to exercise less compared to children without ADHD. Age, financial situation, exposure to greenspace, greenspace perception, and neighbourhood social cohesion were all similar across children with and without ADHD.

**Table 1:** Analytic sample characteristics. Note: *n (%), ** Mean ± standard deviation (minimum – maximum).

Variable	All (n = 679)	Children with ADHD (n = 203 (29.9 %))	Children without ADHD (n = 476 (70.1 %))
**Sociodemographics**			
Town size*			
Small	333 (49.0)	89 (43.8)	244 (51.3)
Large	346 (51.0)	114 (56.2)	232 (48.7)
Sex*			
Female	280 (41.2)	51 (25.1)	229 (48.1)
Male	399 (58.8)	152 (74.9)	247 (51.9)
Age, years**	11.3 ± 0.8 (9.2–14.0)	11.2 ± 0.9 (9.9–14.0)	11.3 ± 0.8 (9.2–13.4)
Parent's minimum education*			
Low	119 (17.5)	49 (24.1)	70 (14.7)
Medium	281 (41.4)	80 (39.4)	201 (42.2)
High	279 (41.1)	74 (36.5)	205 (43.1)
Perceived financial situation*			
Very difficult/quite difficult/we just managed to make ends meet	64 (9.4)	27 (13.3)	37 (7.8)
Doing alright	416 (61.3)	123 (60.6)	293 (61.6)
Living comfortably	177 (26.1)	45 (22.2)	132 (27.7)
Missing	22 (3.2)	8 (3.9)	14 (2.9)
**Exposures**			
Grass/shrub 500 m, %**	33 ± 19.6 (1.2–92.8)	31.9 ± 20.1 (1.2–88.4)	33.4 ± 19.4 (1.8–92.8)
Tree 500 m, %**	9.8 ± 11.2 (0.0–75.6)	9.8 ± 11.5 (0.0–74.7)	9.8 ± 11.1 (0.0–75.6)
Grass/shrub 1 km, %**	36.8 ± 17.9 (2.9–91.1)	35.7 ± 18.4 (5.6–84.6)	37.3 ± 17.7 (2.9–91.1)
Tree 1 km, %**	12.8 ± 12.5 (0.4–67.8)	12.5 ± 11.3 (0.8–61.9)	12.9 ± 13.0 (0.4–67.8)
Garden*			
Yes	408 (60.1)	107 (52.7)	301 (63.2)
No	271 (39.9)	96 (47.3)	175 (36.8)
**Potential mediators**			
Greenspace perception**	5.9 ± 0.9 (1.0–7.0)	5.7 ± 1.0 (2.0–7.0)	5.9 ± 0.9 (1.0–7.0)
Neighbourhood cohesion**	47.4 ± 10.9 (9.0–63.0)	46.3 ± 10.8 (9.0–63.0)	47.9 ± 10.9 (9.0–63.0)
Physical activity duration*			
None	92 (13.6)	45 (22.2)	47 (9.9)
< 1 h/week	74 (10.9)	20 (9.9)	54 (11.3)
1–2 h/week	177 (26.1)	50 (24.6)	127 (26.7)
3–4 h/week	172 (25.3)	52 (25.6)	120 (25.2)
5–6 h/week	92 (13.6)	26 (12.8)	66 (13.9)
> 6 h/week	72 (10.6)	10 (4.9)	62 (13.0)
Physical activity frequency*			
Never	67 (9.9)	34 (16.8)	33 (6.9)
< 1/month	53 (7.8)	19 (9.4)	34 (7.1)
1/month	69 (10.2)	21 (10.3)	48 (10.1)
1–3/week	370 (54.5)	99 (48.8)	271 (56.9)
4–6/week	83 (12.2)	25 (12.3)	58 (12.2)
Every day	37 (5.5)	5 (2.5)	32 (6.7)
Outcomes**			
Internalizing problems	9.3 ± 7.3 (0.0–38.0)	12.8 ± 7.6 (1.0–38.0)	7.8 ± 6.7 (0.0–36.0)
Externalizing problems	10.7 ± 8.4 (0.0–49.0)	17.4 ± 9.3 (1.0–49.0)	7.9 ± 6.1 (0.0–31.0)
Total problems	34.8 ± 23.4 (0.0–138.0)	53.7 ± 23.5 (7.0–138.0)	26.7 ± 18.1 (0.0–113)

### 3.2 Correlations in the data

**Fig. 3:**
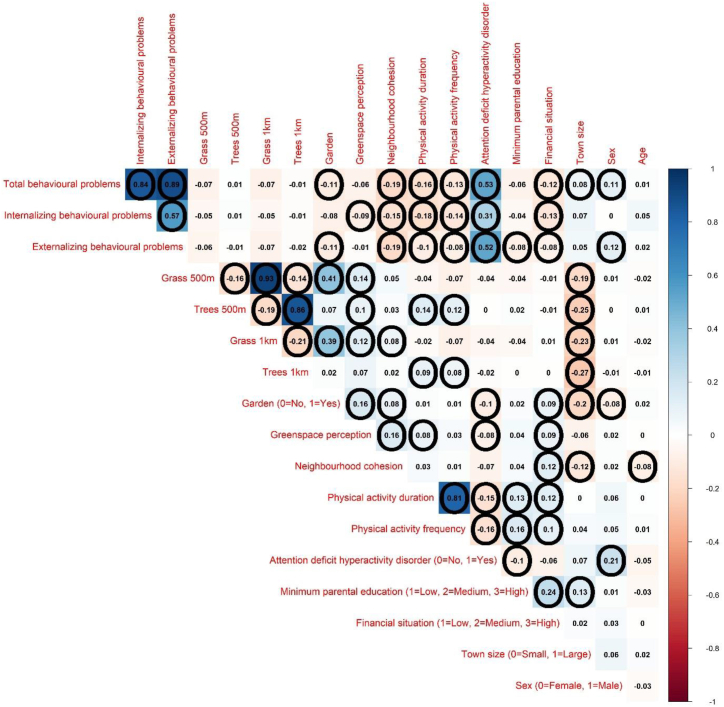
Pairwise correlation coefficients. Pearson correlation coefficients for pairs of numerical variables, including ordinal variables converted to numerical, phi coefficients for pairs of binary variables, and point-biserial correlation coefficients for numerical-binary variable pairs. Encircled correlation coefficients are statistically significant at the 5 % significance level.

Correlations between the variables in the study are shown in Fig. 3. Strong positive correlations were observed between total behavioural problems and internalizing and externalizing behavioural problems, between 500 m and 1000 m buffers of the same type of land-cover-derived greenspace metric, and between physical activity duration and frequency. Moderate positive correlations were seen between ADHD and all types of behavioural problems, between ADHD and being male, and between grass cover and presence of garden. Neighbourhood social cohesion and physical activity were weakly negatively correlated to all types of behavioural problems. Physical activity was weakly positively correlated with tree cover. Town size was weakly negatively correlated with all greenspace metrics. Financial situation was weakly positively correlated with minimum parental education. The remaining correlations were either not significant or small in absolute value.

### 3.3 SEM results

**Fig. 4:**
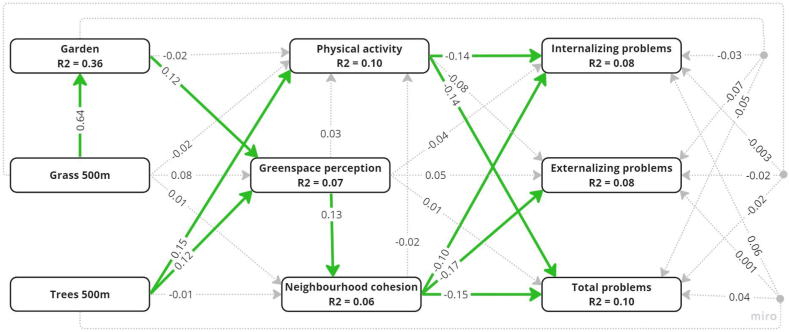
Diagram with path coefficients, estimated in the full sample, of the structural equation modelling (SEM) model for greenspace availability and behavioural problems with mediators physical activity, greenspace perception, and neighbourhood social cohesion and confounders age, sex, socio-economic status (SES), and town size. Coefficients for all but the dichotomous presence of garden are standardized. Green bold lines have path coefficients whose 95 % confidence intervals do not contain zero. R2 shows the proportion of variance explained in each endogenous variable. Confounders, covariances, and error terms are not displayed to enhance readability. (For interpretation of the references to colour in this figure legend, the reader is referred to the Web version of this article.)

The SEM model converged after 71 iterations and fit indices indicated good model fit: χ^2^ = 0.252 (34 degrees of freedom) with p-value >0.05, RMSEA = 0.01 (90 % CI: 0.00, 0.03), SRMR = 0.01, CFI = 0.99, χ2/df = 0.007, TLI = 0.999. Table S2 contains the latent variable loadings. Fig. 4 and Tables S3 and S4 show results from the main SEM model. There was no direct effect of tree cover, grass cover, or garden on internalizing, externalizing, or total behavioural problems. However, we found that some of the examined regression paths were significant. More tree cover was related to more physical activity which, in turn, was related to fewer internalizing and total behavioural problems. More tree cover and presence of garden were related to a higher greenspace perception which, in turn, was related to higher neighbourhood cohesion which, in turn, was related to fewer internalizing, externalizing, and total behavioural problems. Grass cover was not significantly related to any of the mediators. The indirect effect of more tree cover, through increased physical activity, was fewer internalizing and total problems. The indirect effect of more tree cover and presence of garden, through increased greenspace perception and then neighbourhood cohesion, was fewer internalizing, externalizing, and total behavioural problems. Finally, the total indirect (problem-reducing) effect of tree cover on internalizing behavioural problems was also significant.

When stratified by ADHD status, the patterns of associations in children without ADHD were very similar to those observed in the full sample. Two exceptions were the paths ‘garden to greenspace perception’ and ‘physical activity to total behavioural problems’, which were no longer significant (Fig. S4 and Tables S5 and S6). In children with ADHD, only the paths ‘grass cover to garden’ and ‘tree cover to physical activity’ persisted (Fig. S5 and Tables S7 and S8).

When stratified by sex, the patterns of association exhibited notable differences between girls and boys compared to those observed in the entire sample. Among boys, significant associations persisted, particularly between tree cover and physical activity, as well as between tree cover, garden, and greenspace perception (Fig. S6 and Tables S9 and S10). In contrast, Among girls, the only remained significant association was between neighbourhood cohesion and internalizing, externalizing, and total behavioural problems; all other pathways became insignificant (S7 and Tables S11 and S12).

When we increased the size of the buffer around home addresses used for computing tree and grass cover from 500 m to 1 km, the patterns of associations were very similar to those observed with 500 m buffers in the main SEM model, except that the total indirect effect of tree cover did not survive in terms of statistical significance (Fig. S8 and Tables S13 and S14).

## 4 Discussion

### 4.1 Main findings

To our knowledge, this is the second study exploring pathways linking greenspace to behavioural problems in children and the first one to collectively test greenspace perception, physical activity, and neighbourhood cohesion as potential mediators. We analysed data of 679 Polish children aged 10–13 years with and without ADHD. We found that more tree cover was associated with more physical activity which, in turn, was associated with fewer internalizing and total behavioural problems as reported by parents. Furthermore, more tree cover and presence of garden were associated with higher greenspace perception which, in turn, was associated with higher neighbourhood cohesion which, in turn, was associated with fewer internalizing, externalizing, and total behavioural problems. These results were almost entirely replicated in children without ADHD, but not in children with ADHD. When stratified by sex, among boys, more tree cover was associated with more physical activity and more tree cover and presence of garden were associated with higher greenspace perception. Among girls, neighbourhood cohesion was associated with fewer internalizing, externalizing and total behavioural problems.

### 4.2 Comparison with previous research and interpretations

Interest in the connection between greenspace and behavioural problems is not new. A number of studies have examined this relationship using regression analysis, but without estimating potential underlying mechanisms. We have summarized them in Supplemental Table S15. Existing studies (n = 17) were conducted in different regions of the world, with half of them carried out in Western Europe. Study design varied across the studies, with 11 being cross-sectional and 6 longitudinal. The Strengths and Difficulties Questionnaire (SDQ; [50]), a brief screening questionnaire, was the primary tool utilized in the majority of the studies (n = 11) to assess behavioural problems. Only three studies used the more comprehensive CBCL. Most of the studies used either the total behavioural problems score or ‘narrowband’ scores, and only some used ‘broadband’ scores, which limits direct comparisons with published research.

Our study did not reveal a direct effect of tree cover, grass cover, or garden on behavioural problems. However, several previous studies that utilized regression analysis did observe beneficial associations between greenspace and behavioural problems [[51], [52], [53]]; [[54], [55], [56]]; [57,[32], [58], [59], [60]]. Considering studies that used CBCL to measure behavioural problems, in Belgium, Bijnens and colleagues (2020) reported that higher residential greenspace was associated with lower behavioural problems in children aged 7–15 years but only in children living in urban areas. Meanwhile, in China, Liao and colleagues (2020) found that greenness within a circular 100 m buffer around the residence and kindergarten was associated with decreased total behavioural problem in 5- to 6-year-old children. Considering studies that used SDQ, in Germany, Markevych and colleagues (2014) discovered that not having urban green spaces in 500 m distance from home was associated with increased behavioural problems among 10-year-old children. In an Australian study, higher green space quantity and quality were associated with a lower total difficulties score and a lower internalizing subscale score among 12- to 13-year-old children, but only when behavioural problems were reported by parents, not by teachers (Feng et al., 2017a). In England, McEachan and colleagues (2018) studied 4-year-old children and reported that higher green space and satisfaction with green space was associated with fewer internalizing and total behavioural difficulties, but only among south Asian children; no such associations were observed among British children. In the USA, Madzia and colleagues utilized the Behaviour Assessment System for Children, Parent Rating Scale, Second Edition (BASC-2) to measure behavioural problems and reported that increased exposure to greenspace is associated with reduced internalizing and externalizing behavioural problems in 7- and 12-year-old children.

To the best of our knowledge, we are the second study worldwide to investigate potential underlying pathways linking nature to behavioural problems in children. One study conducted in Austria and Italy by Dzhambov and colleagues (2022) explored potential mechanisms of the association between greenspace and behavioural problems in 8- to 12-year-old children. The authors found that children going to schools located close to nature had fever classroom behavioural problems. That study relied on a scale developed by Needleman, rather than a clinical screening tool and expert diagnosis like in the present study. Additionally, the effect of having a home garden acted indirectly by reducing the likelihood of second-hand smoke exposure, which also was associated with fewer behavioural problems.

Due to lack of similar studies and that would explore the same mechanisms and also in children with and without ADHD population, we cannot directly compare our SEM-based results with prior research. In addition, case-control design of our study asked us to an oversampling of children with ADHD in this particular analysis. Nevertheless, SEM results on the children without ADHD were very similar to the results of the analytic sample. Our findings are consistent with the previously established hypotheses and observations, which have not been comprehensively integrated before. In line with prior research, we found that greenspace improves neighbourhood cohesion [28], greenspace perception [27], and physical activity [26] which, in turn, reduce behavioural problems [29,30]. In essence, we confirm findings from existing research.

Tree cover may be of particular interest as many people find trees visually pleasing. Trees provide shade, making the area cooler and more comfortable. Trees often support a greater variety of wildlife compared to grassy areas. Birdwatching, for example, can be more enjoyable in a treed area due to the presence of birds and other creatures [61]. The rustling leaves and the sound of the wind in trees can create a soothing and tranquil atmosphere, making tree-filled areas ideal for relaxation and stress relief. Trees can offer more recreational opportunities, such as tree climbing, setting up hammocks, or using trees as anchors for various outdoor activities. Trees contribute to environmental benefits like improved air quality, carbon sequestration, and habitat for various species. Exposure to different types of greenspace exposure and fewer behavioural problems is under-researched and it is a highly policy-relevant issue, as urban and landscape planners seek evidence on which types of green space offer the strongest overall health gains [62].

In our sample, it is fascinating to observe how different aspects of the neighbourhood play a significant role in the lives of both boys and girls. For boys, trees and gardens seem to have a profound impact, leading to an increase in their physical activity levels, possibly fueled by the allure of outdoor adventures and use of distant neighbourhood greenspace. On the other hand, sense of togetherness and connectedness with their community seems to be relatively more important for girls, influencing a notable decrease in their internalizing, externalizing and total behavioural problems. These gender-specific preferences highlight the diverse ways in which the local environment can influence the well-being of children.

### 4.3 Strengths and limitations

Our study has several notable strengths. Children without ADHD sample was randomly sampled from general population. NeuroSmog provided a comprehensive diagnosis of ADHD using the ICD-11 criteria, which enabled us to examine potentially differential associations between greenspace and behavioural problems in children with ADHD and their peers without ADHD. Behavioural problems were measured with CBCL, a widely used tool with strong validity and reliability [38]. The study utilized geographic information system (GIS) tools to create tree and grass cover variables from high-resolution BDOT10k data. This information is more precise than the satellite-derived normalized difference vegetation index (NDVI) that does not differentiate between vegetation types [24]. Finally, the study included information on physical activity, as well as perceptions of greenspace and cohesion in the neighbourhood, providing a more comprehensive view of the relationship between greenspace exposure and health outcomes through these mediating pathways. We were the first to test the potential mediating role of all these factors in the association of interest.

Although our study provides valuable insights about the psychosocial pathways behind the association between greenspace and behavioural problems, there are several noteworthy limitations. The drawbacks of the retrospective case-control design are well known. For instance, address histories may be affected by recall bias and information on greenspace perception and neighbourhood cohesion may be subject to observer bias. We acknowledge that these factors may have affected the precision of the effect estimates but we do not believe they have produced any directional shift in the results. To achieve an unbiased cognitive evaluation, we adhered to stringent inclusion and exclusion criteria, and did not enrol children with severe morbidities. Also, individuals from immigrant families are underrepresented in NeuroSmog because only Polish-speaking families were recruited. Both of these issues limit the generalizability of our findings to the general population and to the population of children with ADHD. Even though we adjusted for two proxies of personal-level SES, we did not have data on area-level SES, which is a source of residual confounding. While we were able to capture overall physical activity levels and their relationship with nature availability, the absence of physical activity location data limits our understanding of how preciously nature affect physical activity patterns. This limitation underscores the need for future research to capture such data for a more comprehensive understanding. Our findings could have been due to other, non-measured factors. For instance, both higher greenspace perception and higher neighbourhood social cohesion might be manifestations of living in a more affluent neighbourhood. It could be that the affluence itself, rather than perceptions of greenspace or cohesion, leads to fewer behavioural problems. When interpreting our results, it should be kept in mind that, except for objectively assessed grass and tree cover, all data were parent-reported. We do not have data from the child's perspective.

### 4.4 Implications and recommendations for future research

Rapid urbanisation in Europe has resulted in poor availability and accessibility of greenspaces in many cities, which comes with a significant health burden [[63], [64]]. According to the ISGlobal city ranking, which evaluates the environmental quality and sustainable development of cities around the world, several assessed Polish cities rank low in terms of greenspace availability [65].

Several strategies could be implemented to increase greenspace availability and accessibility in urban areas, such as creating new parks, even if they are only small pocket parks, planting more trees, adding green walls, flowerbeds, and bushes, and promoting urban agriculture. These strategies could provide numerous physical and mental health benefits, reduce air pollution, and increase cohesion. Greenspace, such as forests and parks, also plays a significant role in promoting biodiversity and mitigating climate change by absorbing carbon dioxide and reducing the effect of the urban heat island effect [66,67].

Future research should go beyond cross-sectional regression analyses and tackle the trajectories of mental health development via longitudinal study designs that allow causal inference. More studies are needed to replicate our findings, also in other climatic and cultural settings. There are many other potential mechanisms which can play a role, beyond what we tested, and they should be explored, for instance, air pollution such as NO_2_ [68] and PM_2.5_ [69,[70], [71]], and biodiversity [72]. CBCL should be preferred to the less comprehensive SDQ. Collecting data from different respondents can also be informative. For example, externalizing behavioural problems might be better seen by teachers than parents, while parents might be more observant about internalizing problems. Finally, the association of interest should be investigated in clinical populations, such as children with ADHD.

### 4.5 Conclusions

Polish children present fewer behavioural problems when they are surrounded by trees or have a home garden in their living environment. This association operates through higher greenspace perception, a greater sense of community, and increased physical activity levels. Grass is not important in this context.

## Funding

Supported by “NeuroSmog: Determining the impact of air pollution on the developing brain” (Nr. POIR.04.04.00-00-1763/18) grant to Marcin Szwed implemented as part of the TEAM-NET programme of the Foundation for Polish Science, co-financed from EU resources obtained from the European Regional Development Fund under the Smart Growth Operational Programme. The aforementioned funding sources had no involvement in the design of the study, collection, analysis nor interpretation of the data, writing of the report, nor in the decision to submit the manuscript for publication. Angel Dzhambov's and Iana Markevych's time on this publication is partially supported by the “Strategic research and innovation program for the development of Medical University – Plovdiv” № BG-RRP-2.004-0007-C01, Establishment of a network of research higher schools, National plan for recovery and resilience, financed by the 10.13039/501100000780European Union – NextGenerationEU".

## Data availability statement

Data were obtained from the NeuroSmog study, which is bound to the local ethical and legal restrictions with respect to the study data. The informed consent provided by the NeuroSmog study participants did not include data posted in public databases. However, all data used for this publication are available upon request. Contact person is Dr Iana Markevych (iana.markevych@uj.edu.pl).

## CRediT authorship contribution statement

**Nitika Singh:** Writing – review & editing, Writing – original draft, Visualization, Software, Methodology, Formal analysis, Conceptualization. **Dorota Buczyłowska:** Writing – review & editing, Supervision, Data curation, Conceptualization. **Clemens Baumbach:** Writing – review & editing, Supervision, Software, Formal analysis, Data curation, Conceptualization. **Jakub Bratkowski:** Writing – review & editing, Software, Resources. **Yarema Mysak:** Writing – review & editing, Data curation. **Maja Wierzba-Łukaszyk:** Writing – review & editing, Data curation. **Katarzyna Sitnik-Warchulska:** Writing – review & editing, Resources, Data curation. **Krzysztof Skotak:** Writing – review & editing, Resources. **Małgorzata Lipowska:** Writing – review & editing, Resources, Data curation. **Bernadetta Izydorczyk:** Writing – review & editing, Resources, Data curation. **Marcin Szwed:** Writing – review & editing, Resources, Funding acquisition. **Angel M. Dzhambov:** Writing – review & editing, Supervision, Conceptualization. **Iana Markevych:** Writing – review & editing, Writing – original draft, Supervision, Resources, Data curation, Conceptualization.

## Declaration of competing interest

The authors declare that they have no known competing financial interests or personal relationships that could have appeared to influence the work reported in this paper.

## References

[1] Reef J., Diamantopoulou S., Van Meurs I., Verhulst F.C., Van Der Ende J. (2011). Developmental trajectories of child to adolescent externalizing behavior and adult DSM-IV disorder: results of a 24-year longitudinal study. Soc. Psychiatr. Psychiatr. Epidemiol..

[2] Lahey B.B. (2015). Why are children who exhibit psychopathology at high risk for psychopathology and dysfunction in adulthood?. JAMA Psychiatr..

[3] Hammerton G., Murray J.A., Maughan B., Barros F.C., Gonçalves H., Hallal P.C., Wehrmeister F.C., Hickman M., Heron J. (2019). Childhood behavioural problems and adverse outcomes in early adulthood: a comparison of Brazilian and British birth cohorts. Journal of Developmental and Life-Course Criminology.

[4] Achenbach T.M. (2009).

[5] Achenbach T.M., Rescorla L.A. (2007).

[6] World Health Organization (WHO) (2021). Comprehensive mental health action plan 2013–2030.

[7] Kaplan R., Kaplan S. (1989).

[8] Kaplan S. (1995). The restorative benefits of nature: toward an integrative framework. J. Environ. Psychol..

[9] Ulrich R.K., Simons R.A., Losito B.D., Fiorito E., Miles M., Zelson M.F. (1991). Stress recovery during exposure to natural and urban environments. J. Environ. Psychol..

[10] Yang B., Zhao T., Hu L., Browning M.H.E.M., Heinrich J., Dharmage S.C., Jalaludin B., Knibbs L.D., Liu X., Luo Y., James P., Li S., Huang W., Chen G., Zeng X., Yu Y., Dong G. (2021). Greenspace and human health: an umbrella review. Innovation.

[11] Tyrväinen L., Ojala A., Korpela K., Lanki T., Tsunetsugu Y., Kagawa T. (2014). The influence of urban green environments on stress relief measures: a field experiment. J. Environ. Psychol..

[12] Coventry P.A., Brown J.E., Pervin J., Brabyn S., Pateman R., Breedvelt J., Gilbody S., Stancliffe R., McEachan R., White P.L. (2021). Nature-based outdoor activities for mental and physical health: systematic review and meta-analysis. SSM - Population Health.

[13] Bratman G.N., Hamilton J.H., Hahn K.A., Daily G.C., Gross J.J. (2015). Nature experience reduces rumination and subgenual prefrontal cortex activation. Proc. Natl. Acad. Sci. U.S.A..

[14] Bratman G.N., Young G., Mehta A., Lee Babineaux I., Daily G.C., Gross J.J. (2021). Affective benefits of nature contact: the role of rumination. Front. Psychol..

[15] Kotera Y., Lyons M., Vione K.C., Norton B. (2021). Effect of nature walks on depression and anxiety: a systematic review. Sustainability.

[16] Madzia J., Ryan P.B., Yolton K., Percy Z., Newman N., LeMasters G.K., Price-Whelan A.M. (2019). Residential greenspace association with childhood behavioral outcomes. J. Pediatr..

[17] Shin J.H., Parab K.V., An R., Grigsby-Toussaint D.S. (2020). Greenspace exposure and sleep: a systematic review. Environ. Res..

[18] Jimenez M.P., DeVille N.V., Elliott E.G., Schiff J.E., Wilt G.E., Hart J.E., James P. (2021). Associations between nature exposure and health: a review of the evidence. Int. J. Environ. Res. Publ. Health.

[19] Nejade R.M., Grace D., Bowman L.R. (2022). What is the impact of nature on human health? A scoping review of the literature. Journal of Global Health.

[20] Vella-Brodrick D., Gilowska K. (2022). Effects of nature (greenspace) on cognitive functioning in school children and adolescents: a systematic review. Educ. Psychol. Rev..

[21] Zelenski J.M., Nisbet E.K. (2014). Happiness and feeling connected: the distinct role of nature relatedness. Environ. Behav..

[22] Zhang J., Piff P.K., Iyer R., Koleva S., Keltner D. (2014). An occasion for unselfing: beautiful nature leads to prosociality. J. Environ. Psychol..

[23] Ratcliffe E., Gatersleben B., Sowden P.T. (2013). Bird sounds and their contributions to perceived attention restoration and stress recovery. J. Environ. Psychol..

[24] Markevych I., Schoierer J., Hartig T., Chudnovsky A., Hystad P., Dzhambov A.M., De Vries S., Triguero-Mas M., Brauer M., Nieuwenhuijsen M.J., Lupp G., Richardson E.A., Astell-Burt T., Dimitrova D.D., Feng X., Sadeh M., Standl M., Heinrich J., Fuertes E. (2017). Exploring pathways linking greenspace to health: theoretical and methodological guidance. Environ. Res..

[25] Kuo M., Barnes M.R., Jordan C.M. (2019). Do experiences with nature promote learning? Converging evidence of a cause-and-effect relationship. Front. Psychol..

[26] Armstrong G.P., Maitland C., Lester L., Trost S.G., Trapp G., Boruff B., Marzooqi M.K.A., Christian H. (2019). Associations between the home yard and preschoolers' outdoor play and physical activity. Public Health Research & Practice.

[27] Reid C.E., Rieves E., Carlson K. (2022). Perceptions of green space usage, abundance, and quality of green space were associated with better mental health during the COVID-19 pandemic among residents of Denver. PLoS One.

[28] Jennings V., Bamkole O. (2019). The relationship between social cohesion and urban green space: an avenue for health promotion. Int. J. Environ. Res. Publ. Health.

[29] Kuo M. (2015). How might contact with nature promote human health? Promising mechanisms and a possible central pathway. Front. Psychol..

[30] Sakhvidi M.J.Z., Knobel P., Bauwelinck M., De Keijzer C., Boll L.M., Spano G., Ubalde-Lopez M., Sanesi G., Mehrparvar A.H., Jacquemin B., Dadvand P. (2022). Greenspace exposure and children behavior: a systematic review. Sci. Total Environ..

[31] Pearson A.L., Brown C.D., Reuben A., Nicholls N., Pfeiffer K.A., Clevenger K.A. (2023). Elementary classroom views of nature are associated with lower child externalizing behavior problems. Int. J. Environ. Res. Publ. Health.

[32] Dzhambov A.M., Lercher P., Tappeiner U., Browning M.H.E.M., Markevych I. (2022). Home gardens and distances to nature associated with behavior problems in alpine schoolchildren: role of secondhand smoke exposure and biomarkers. Int. J. Hyg Environ. Health.

[33] Taylor A.F., Kuo F.E. (2011). Could exposure to everyday green spaces help treat ADHD? Evidence from children's play settings. Appl. Psychol.: Health and Well-being.

[34] World Health Organization (WHO) (2019). Attention deficit hyperactivity disorder (ADHD).

[35] Markevych I., Orlov N., Grellier J., Kaczmarek-Majer K., Lipowski M., Sitnik-Warchulska K., Mysak Y., Baumbach C., Wierzba-Łukaszyk M., Soomro M.H., Compa M., Izydorczyk B., Skotak K., Degórska A., Bratkowski J., Kossowski B., Domagalik A., Szwed M. (2022). NeuroSmog: determining the impact of air pollution on the developing brain: project protocol. Int. J. Environ. Res. Publ. Health.

[36] Achenbach T.M., Rescorla L.A. (2001).

[37] Wolańczyk T. (2002).

[38] Nakamura B.J., Ebesutani C., Bernstein A. (2009). A psychometric analysis of the child behavior checklist DSM-oriented scales. J. Psychopathol. Behav. Assess..

[39] Albores-Gallo L., Lara-Muñoz C., Esperón-Vargas C., Zetina J.A.C., Soriano A.M.P., Colin G.V. (2007). Validity and reliability of the CBCL/6-18. Includes DSM scales. Actas Esp. Psiquiatr..

[40] Twohig-Bennett C., Jones A.M. (2018). The health benefits of the great outdoors: a systematic review and meta-analysis of greenspace exposure and health outcomes. Environ. Res..

[41] Astell-Burt T., Walsan R., Davis W., Feng X. (2022). What types of green space disrupt a lonelygenic environment? A cohort study. Soc. Psychiatr. Psychiatr. Epidemiol..

[42] Feng X., Navakatikyan M.A., Toms R., Astell-Burt T. (2022). Leafier communities, healthier hearts: an Australian cohort study of 104,725 adults tracking cardiovascular events and mortality across 10 Years of linked health data. Heart Lung Circ..

[43] Singh N., Baumbach C., Buczyłowska D., Bratkowski J., Mysak Y., Wierzba-Łukaszyk M., Sitnik-Warchulska K., Skotak K., Lipowska M., Izydorczyk B., Szwed M., Markevych I. (2023). Association of residential and school green- and bluespace with academic performance in 10-13-year-old Polish schoolchildren with and without attention deficit hyperactivity disorder. Sci. Total Environ..

[44] Dupuis M., Baggio S., Gmel G. (2017). Validation of a brief form of the perceived neighbourhood social cohesion questionnaire. J. Health Psychol..

[45] Hellström-Lindberg E., Janson C., Johannessen A., Svanes C., Real F.X., Malinovschi A., Franklin K.A., Holm M., Schlünssen V., Jõgi N.O., Gislason T., Benediktsdottir B. (2020). Sleep time and sleep-related symptoms across two generations – results of the community-based RHINE and RHINESSA studies. Sleep Med..

[46] Ekström M., Johannessen A., Abramson M.J., Benediktsdottir B., Franklin K.A., Gislason T., Real F.X., Holm M., Janson C., Jõgi R., Lowe A.J., Malinovschi A., Martínez-Moratalla J., Oudin A., Sánchez-Ramos J.L., Schlünssen V., Svanes C. (2022). Breathlessness across generations: results from the RHINESSA generation study. Thorax.

[47] Ratner B. (2009). The correlation coefficient: its values range between +1/−1, or do they?. J. Target Meas. Anal. Market..

[48] Mitchell C., Clark A., Gilliland J. (2016). Built environment influences of children's physical activity: examining differences by neighbourhood size and sex. Int. J. Environ. Res. Publ. Health.

[49] Marzi I., Demetriou Y., Reimers A.K. (2018). Social and physical environmental correlates of independent mobility in children: a systematic review taking sex/gender differences into account. Int. J. Health Geogr..

[50] Goodman R. (1997). The strengths and difficulties questionnaire: a research note. JCPP (J. Child Psychol. Psychiatry).

[51] Markevych I., Tiesler C.M.T., Fuertes E., Romanos M., Dadvand P., Nieuwenhuijsen M.J., Berdel D., Koletzko S., Heinrich J. (2014). Access to urban green spaces and behavioural problems in children: results from the GINIplus and LISAplus studies. Environ. Int..

[52] Richardson E.A., Pearce J., Shortt N.K., Mitchell R.N. (2017). The role of public and private natural space in children's social, emotional and behavioural development in Scotland: a longitudinal study. Environ. Res..

[53] Amoly E., Dadvand P., Forns J., López-Vicente M., Basagaña X., Julvez J., Alvarez-Pedrerol M., Nieuwenhuijsen M.J., Sunyer J. (2014). Green and blue spaces and behavioral development in barcelona schoolchildren: the BREATHE project. Environ. Health Perspect..

[54] McEachan R.R.C., Yang T., Roberts H., Pickett K.E., Arseneau-Powell D., Gidlow C., Wright J., Nieuwenhuijsen M.J. (2018). Availability, use of, and satisfaction with green space, and children's mental wellbeing at age 4 years in a multicultural, deprived, urban area: results from the Born in Bradford cohort study. Lancet Planet. Health.

[55] Bijnens E.M., Derom C., Thiery E., Weyers S., Nawrot T.S. (2020). Residential green space and child intelligence and behavior across urban, suburban, and rural areas in Belgium: a longitudinal birth cohort study of twins. PLoS Med..

[56] Liao J., Yang S., Xia W., Peng A., Zhao J., Li Y., Zhang Y.D., Qian Z., Vaughn M.G., Schootman M., Zhang B., Xu S. (2020). Associations of exposure to green space with problem behaviours in preschool-aged children. Int. J. Epidemiol..

[57] Jimenez M.P., Aris I.M., Rifas-Shiman S.L., Young J.E., Tiemeier H., Hivert M., Oken E., James P. (2021). Early life exposure to greenness and executive function and behavior: an application of inverse probability weighting of marginal structural models. Environ. Pollut..

[58] Dockx Y., Bijnens E.M., Luyten L.J., Peusens M., Provost E.B., Rasking L., Sleurs H., Hogervorst J.G.F., Plusquin M., Casas L., Nawrot T.S. (2022). Early life exposure to residential green space impacts cognitive functioning in children aged 4 to 6 years. Environ. Int..

[59] Feng X., Astell-Burt T. (2017). The relationship between neighbourhood green space and child mental wellbeing depends upon whom you ask: multilevel evidence from 3083 children aged 12–13 years. Int. J. Environ. Res. Publ. Health.

[60] Feng X., Astell-Burt T. (2017). Residential green space quantity and quality and child well-being: a longitudinal study. Am. J. Prev. Med..

[61] Turner-Skoff J.B., Cavender N. (2019). The benefits of trees for livable and sustainable communities. Plants, People, Planet.

[62] Wolf K.L., Lam S.T., McKeen J.K., Richardson G.R.A., van den Bosch M., Bardekjian A.C. (2020). Urban trees and human health: a scoping review. Int. J. Environ. Res. Publ. Health.

[63] Eurostat, Urban Europe: Statistics on cities, towns and suburbs (2016). https://ec.europa.eu/eurostat/documents/3217494/7596823/KS-01-16-691-EN-N.pdf/0abf140c-ccc7-4a7f-b236-682effcde10f?t=1472645220000. (Accessed 4 May 2023, Pages 7 - 12, ISBN 978-92-79-60139-2, ISSN 2363-1716, doi: 10.2785/91120.

[64] World Bank Group (2019). Country partnership framework for Poland for the period FY20-FY24.

[65] Barboza E.P., Cirach M., Khomenko S., Iungman T., Mueller N., Barrera-Gómez J., Rojas-Rueda D., Kondo M.C., Nieuwenhuijsen M.J. (2021). Green space and mortality in European cities: a health impact assessment study. Lancet Planet. Health.

[66] Palmer L. (2021). How trees and forests reduce risks from climate change. Nat. Clim. Change.

[67] Kong X., Zhang X., Xu C., Hauer R.N. (2021). Review on urban forests and trees as nature-based solutions over 5 years. Forests.

[68] Loftus C.T., Ni Y., Szpiro A.A., Hazlehurst M.F., Tylavsky F.A., Bush N.R., Sathyanarayana S., Carroll K.N., Young M., Karr C.J., LeWinn K.Z. (2020). Exposure to ambient air pollution and early childhood behavior: a longitudinal cohort study. Environ. Res..

[69] Ni Y., Loftus C.T., Szpiro A.A., Young M.T., Hazlehurst M.F., Murphy L.E., Tylavsky F.A., Mason W.A., LeWinn K.Z., Sathyanarayana S., Barrett E.S., Bush N.R., Karr C.J. (2022). Associations of pre- and postnatal air pollution exposures with child behavioral problems and cognitive performance: a U.S. Multi-cohort study. Environ. Health Perspect..

[70] Campbell C.E., Cotter D.L., Bottenhorn K.L., Burnor E., Ahmadi H., Gauderman W.J., Cardenas-Iniguez C., Hackman D., McConnell R., Berhane K., Schwartz J., Chen J.-C., Herting M.M. (2023). Air pollution and emotional behavior in adolescents across the U.S. medRxiv, 2023.04.19.23288834.

[71] Hjortebjerg D., Andersen A.M., Christensen J.S., Ketzel M., Raaschou-Nielsen O., Sunyer J., Julvez J., Forns J., Sørensen M. (2016). Exposure to road traffic noise and behavioral problems in 7-year-old children: a cohort study. Environ. Health Perspect..

[72] Goncalves P., Grilo F., Mendes R., Vierikko K., Elands B., Marques T., Santos‐Reis M. (2021). What's biodiversity got to do with it? Perceptions of biodiversity and restorativeness in urban parks. Ecol. Soc..

