# Supplementary material for: Pathways linking greenspace to behavioural problems in Polish children
Source: Heliyon. 2024 May 18;10(10):e31435. doi: 10.1016/j.heliyon.2024.e31435 (PMC11137514; doi:10.1016/j.heliyon.2024.e31435)
Supplement: Multimedia component 1 [file mmc1.docx]

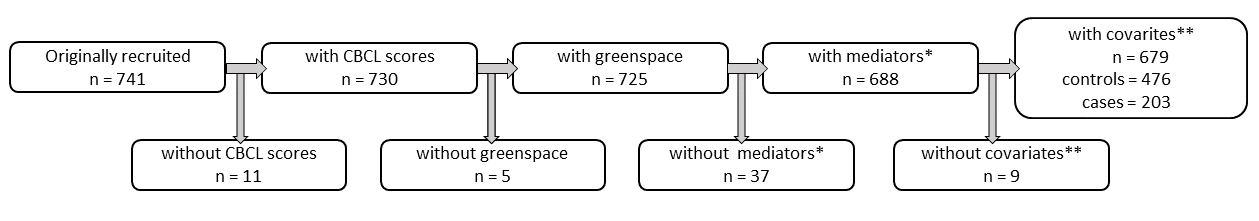


Figure S1: Analytic sample flow diagram.

*Note.* CBCL: Child Behaviour Checklist

*Mediators are greenspace perception, neighbourhood social cohesion and physical activity.

**Covariates are age, sex, parental education, financial situation, and town size.

Figure S2: Behavioral problems measured by Child Behaviour Checklist (CBCL).


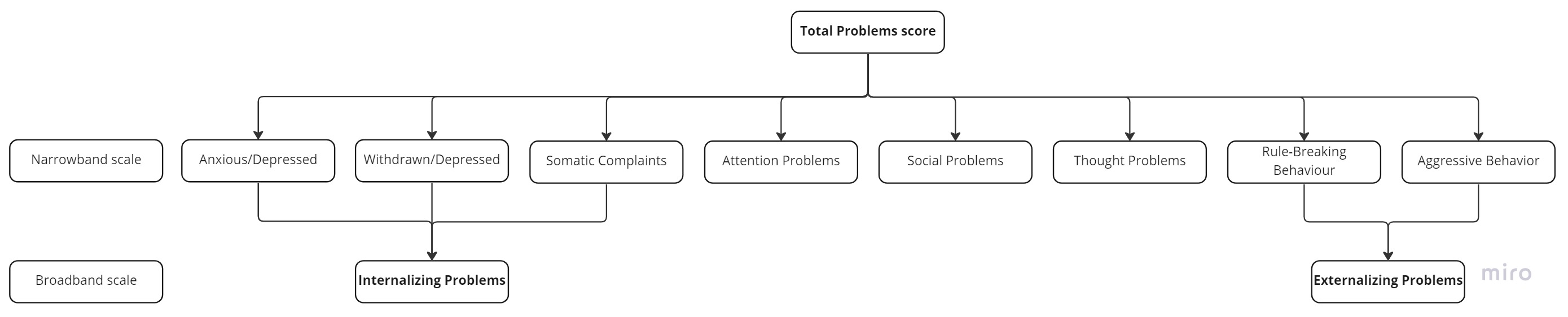


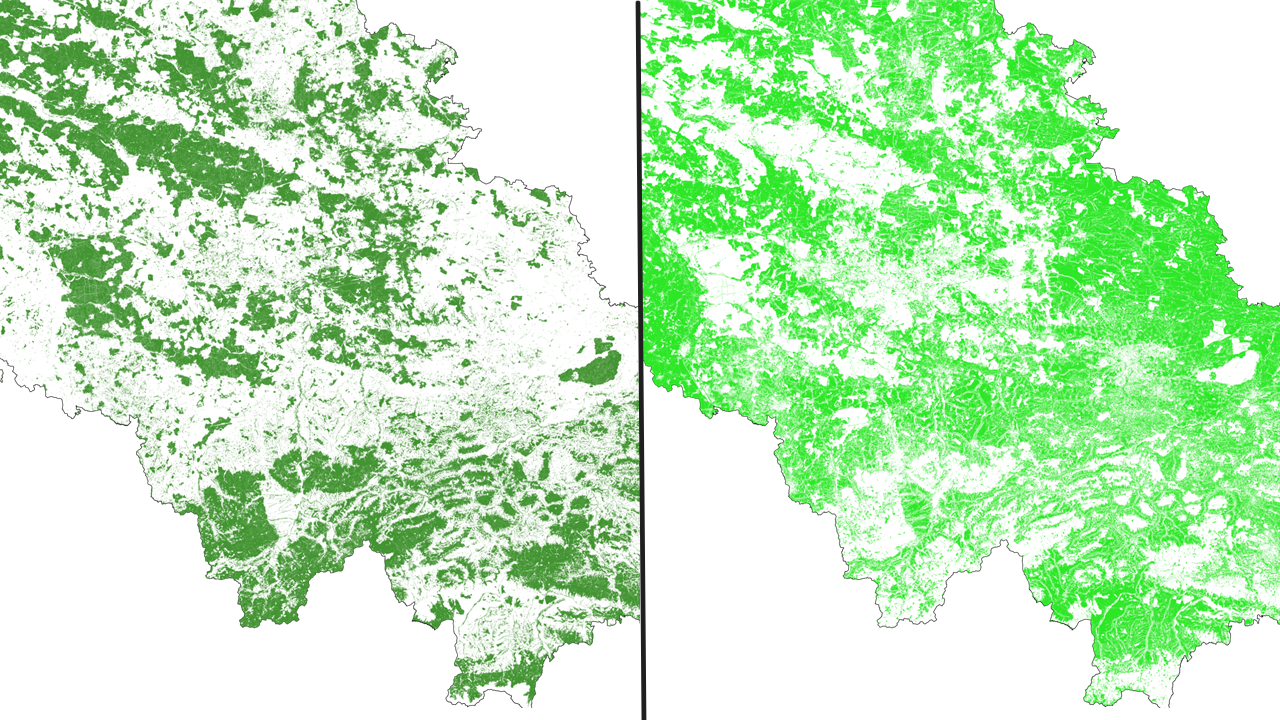


Figure S3. Tree cover (left) and grass cover (right) in the study area.

Table S1: Comparison of included and excluded sample. Note: * n (%), ** N(Mean ± standard deviation)

| Variable | Included N(%) | Excluded N(%) | Pval |
| --- | --- | --- | --- |
| **Sociodemographics** |  |  |  |
| ADHD | 679 | 56 | 0.24 |
| Control | 476(70.1) | 44(78.57) |  |
| Case | 203(29.9) | 12(21.43) |  |
| Town_size* | 679 | 62 | 0.99 |
| Small | 333(49.04) | 31(50) |  |
| Large | 346(50.96) | 31(50) |  |
| Sex* | 679 | 61 | 0.29 |
| Female | 280(41.24) | 30(49.18) |  |
| Male | 399(58.76) | 31(50.82) |  |
| Age** | 679(11.27 ± 0.80) | 61(11.41 ± 0.83) | 0.20 |
| Parent’s minimum education* | 679 | 56 | 0.25 |
| Low | 119(17.53) | 10(17.86) |  |
| Medium | 281(41.38) | 29(51.79) |  |
| High | 279(41.09) | 17(30.36) |  |
| Perceived financial situation* | 679 | 62 | 0.23 |
| Difficult | 64(9.43) | 7(11.29) |  |
| OK | 416(61.27) | 41(66.13) |  |
| Comfy | 177(26.07) | 10(16.13) |  |
| Miss | 22(3.24) | 4(6.45) |  |
| **Exposures** |  |  |  |
| Grass/shrub 500m, %** | 679(32.98 ± 19.62) | 57(37.88 ± 18.59) | 0.06 |
| Tree 500m, %** | 679(9.83 ± 11.20) | 57(9.62 ± 7.84) | 0.85 |
| Garden | 679 | 59 | 0.31 |
| No | 271(39.91) | 19(32.2) |  |
| Yes | 408(60.09) | 40(67.8) |  |
| **Potential mediators**** |  |  |  |
| Greenspace perception | 679(5.86 ± 0.92) | 61(5.72 ± 1.25) | 0.41 |
| Neighbourhood cohesion | 679(47.43 ± 10.87) | 61(43.92 ± 10.35) | 0.01 |
| **Outcomes**** |  |  |  |
| Internalizing problems | 679(9.29 ± 7.34) | 52(10.21 ± 9.22) | 0.48 |
| Externalizing problems | 679(10.73 ± 8.42) | 52(9.38 ± 6.79) | 0.18 |
| Total problems | 679(34.79 ± 23.41) | 52(33.83 ± 22.75) | 0.77 |

Table S2: Loadings of latent variables

| Latent variable | Variables | Loadings | | | |
| --- | --- | --- | --- | --- | --- |
|  |  | Main model | Stratified by ADHD status | | 1km buffer around home for tree and grass cover |
|  |  |  | With ADHD | Without ADHD |  |
| Socio-economic status | Parental education | 1 | 1 | 1 | 1 |
|  | Financial situation | 1.24 | 1.08 | 1.90 | 1.21 |
| Physical activity | Physical activity duration | 1 | 1 | 1 | 1 |
|  | Physical activity frequency | 0.90 | 0.96 | 0.91 | 0.90 |

Table S3: Direct, indirect, and total effects, estimated in the **full sample**, of the structural equation modelling (SEM) model for greenspace availability and behavioural problems with mediators physical activity, greenspace perception, and neighbourhood social cohesion and confounders age, sex, socio-economic status (SES), and town size. Effect estimates whose 95% confidence intervals do not contain zero are shown in bold.

|  | Estimate (95% Confidence Interval) | | |
| --- | --- | --- | --- |
|  | Internalizing problems | Externalizing problems | Total problems |
| **Direct effects** |  |  |  |
| Grass 500m | -0.003 (-0.10 – 0.11) | -0.02 (-0.13 – 0.09) | -0.02 (-0.13 – 0.09) |
| Tree 500m | 0.06 (-0.02 – 0.14) | 0.001 (-0.07 – 0.09) | 0.04 (-0.04 – 0.12) |
| Garden | -0.03 (-0.15 – 0.08) | -0.07 (-0.19 – 0.04) | -0.05 (-0.17 – 0.06) |
| **Indirect effects via physical activity** |  |  |  |
| Grass 500m Physical activity | 0.003 (-0.01 – 0.02) | 0.002 (-0.01 – 0.01) | 0.003 (-0.01 – 0.02) |
| Tree 500m Physical activity | **-0.02 (-0.04 – -0.01)** | -0.01 (-0.03 – 0.002) | **-0.02 (-0.04 – -0.01)** |
| Garden Physical activity | 0.003 (-0.01 – 0.02) | 0.002 (-0.01 – 0.01) | 0.003 (-0.01 – 0.02) |
| **Indirect effects via greenspace perception** |  |  |  |
| Grass 500m Greenspace perception | -0.003 (-0.01 – 0.01) | 0.004 (-0.003 – 0.02) | 0.001 (-0.01 – 0.01) |
| Tree 500m Greenspace perception | -0.01 (-0.02 – 0.004) | 0.01 (-0.004 – 0.02) | 0.001 (-0.01 – 0.01) |
| Garden Greenspace perception | -0.01 (-0.02 – 0.01) | 0.01 (-0.004 – 0.02) | 0.001 (-0.01 – 0.01) |
| **Indirect effects via neighbourhood cohesion** |  |  |  |
| Grass 500m Neighbourhood cohesion | -0.001 (-0.01 – 0.01) | -0.001 (-0.02 – 0.02) | -0.001 (-0.01 – 0.01) |
| Tree 500m Neighbourhood cohesion | 0.001 (-0.01 – 0.01) | 0.002 (-0.01 – 0.02) | 0.001 (-0.01 – 0.02) |
| **Specific indirect effects** |  |  |  |
| Grass 500m Greenspace perception Physical activity | -0.0004 (-0.002 – 0.001) | -0.0002 (-0.001 – 0.0003) | -0.0004 (-0.002 – 0.001) |
| Trees 500m Greenspace perception Physical activity | -0.001 (-0.003 – 0.001) | -0.0003 (-0.002 – 0.001) | -0.001 (-0.003 – 0.001) |
| Garden Greenspace perception Physical activity | -0.001 (-0.003 – 0.001) | -0.0003 (-0.002 – 0.001) | -0.001 (-0.003 – 0.001) |
| Grass 500m Neighbourhood cohesion Physical activity | 0.00002 (-0.001 – 0.001) | 0.00001 (-0.0004 – 0.0004) | 0.00001 (-0.001 – 0.001) |
| Trees 500m Neighbourhood cohesion Physical activity | -0.00003 (-0.001 – 0.001) | -0.00002 (-0.001 – 0.0003) | -0.00003 (-0.001 – 0.0005) |
| Grass 500m Greenspace perception Neighbourhood cohesion | -0.001 (-0.003 – 0.0004) | -0.002 (-0.005 – 0.001) | -0.002 (-0.004 – 0.001) |
| Trees 500m Greenspace perception Neighbourhood cohesion | **-0.002 (-0.004 – -0.0002)** | **-0.003 (-0.01 – -0.001)** | **-0.002 (-0.01 – -0.001)** |
| Garden Greenspace perception Neighbourhood cohesion | **-0.002 (-0.01 – -0.0001)** | **-0.003 (-0.01 – -0.0004)** | **-0.002 (-0.01 – -0.0003)** |
| **Total indirect effects** |  |  |  |
| Grass 500m | -0.001 (-0.02 – 0.02) | 0.005 (-0.02 – 0.03) | 0.003 (-0.02 – 0.03) |
| Trees 500m | **-0.03 (-0.05 – -0.003)** | -0.004 (-0.03 – 0.02) | -0.02 (-0.05 – 0.01) |
| Garden | -0.002 (-0.02 – 0.02) | 0.01 (-0.01 – 0.03) | 0.004 (-0.02 – 0.02) |
| **Total effects** |  |  |  |
| Grass 500m | -0.003 (-0.11 – 0.11) | -0.01 (-0.12 – 0.10) | -0.02 (-0.13 – 0.10) |
| Trees 500m | 0.03 (-0.04 – 0.10) | -0.003 (-0.08 – 0.08) | 0.02 (-0.06 – 0.10) |
| Garden | -0.04 (-0.15 – 0.08) | -0.06 (-0.18 – 0.04) | -0.05 (-0.17 – 0.07) |

Table S4: Path coefficients and 95% bootstrap confidence intervals (CI), estimated in the **full sample**, of the structural equation modelling (SEM) model for greenspace availability and behavioural problems with mediators physical activity, greenspace perception, and neighbourhood social cohesion and confounders age, sex, socio-economic status (SES), and town size. Estimates whose 95% CIs do not contain zero are shown in bold.

| Estimate (95% Confidence Interval) | | | | | | |  |
| --- | --- | --- | --- | --- | --- | --- | --- |
|  | Internalizing problems ~ | Externalizing problems ~ | Total  problems ~ | Physical  activity ~ | Greenspace  perception ~ | Neighbourhood cohesion ~ | Garden ~ |
| Grass 500m | -0.003 (-0.10 – 0.11) | -0.02 (-0.13 – 0.09) | -0.02 (-0.13 – 0.09) | -0.02 (-0.14 – 0.09) | 0.08 (-0.03 – 0.17) | 0.01 (-0.08 – 0.08) | **0.64 (0.52 – 0.79)** |
| Trees 500m | 0.06 (-0.02 – 0.14) | 0.001 (-0.07 – 0.09) | 0.04 (-0.04 – 0.12) | **0.15 (0.07 – 0.23)** | **0.12 (0.05 – 0.20)** | -0.01 (-0.10 – 0.06) |  |
| Garden | -0.03 (-0.15 – 0.08) | -0.07 (-0.19 – 0.04) | -0.05 (-0.17 – 0.06) | -0.02 (-0.14 – 0.09) | **0.12 (0.02 – 0.23)** |  |  |
| Greenspace perception | -0.04 (-0.13 – 0.05) | 0.05 (-0.03 – 0.13) | 0.01 (-0.07 – 0.09) | 0.03 (-0.05 – 0.12) |  | **0.13 (0.06 – 0.20)** |  |
| Neighbourhood cohesion | **-0.10 (-0.18 – -0.02)** | **-0.17 (-0.24 – -0.09)** | **-0.15 (-0.23 – -0.08)** | -0.02 (-0.10 – 0.07) |  |  |  |
| Physical activity | **-0.14 (-0.25 – -0.05)** | -0.08 (-0.18 – 0.02) | **-0.14 (-0.24 – -0.04)** |  |  |  |  |
| Socio-economic status | -0.40 (-0.98 – 0.07) | -0.36 (-0.88 – 0.09) | -0.43 (-1.03 – 0.02) | **0.88 (0.44 – 1.46)** | 0.39 (-0.02 – 0.86) | **0.58 (0.18 – 1.13)** | **0.84 (0.27 – 1.59)** |
| Age | 0.05 (-0.03 – 0.13) | 0.02 (-0.06 – 0.10) | 0.01 (-0.07 – 0.09) | 0.01 (-0.07 – 0.09) | -0.01 (-0.08 – 0.06) |  |  |
| Sex | 0.01 (-0.15 – 0.16) | **0.24 (0.09 – 0.39)** | **0.23 (0.09 – 0.37)** | 0.13 (-0.03 – 0.28) | 0.03 (-0.11 – 0.19) |  |  |
| Town size | 0.13 (-0.03 – 0.29) | 0.02 (-0.14 – 0.17) | 0.09 (-0.07 – 0.25) | 0.08 (-0.11 – 0.26) | 0.02 (-0.13 – 0.18) | **-0.23 (-0.39 – -0.09)** | **-0.28 (-0.51 – -0.05)** |


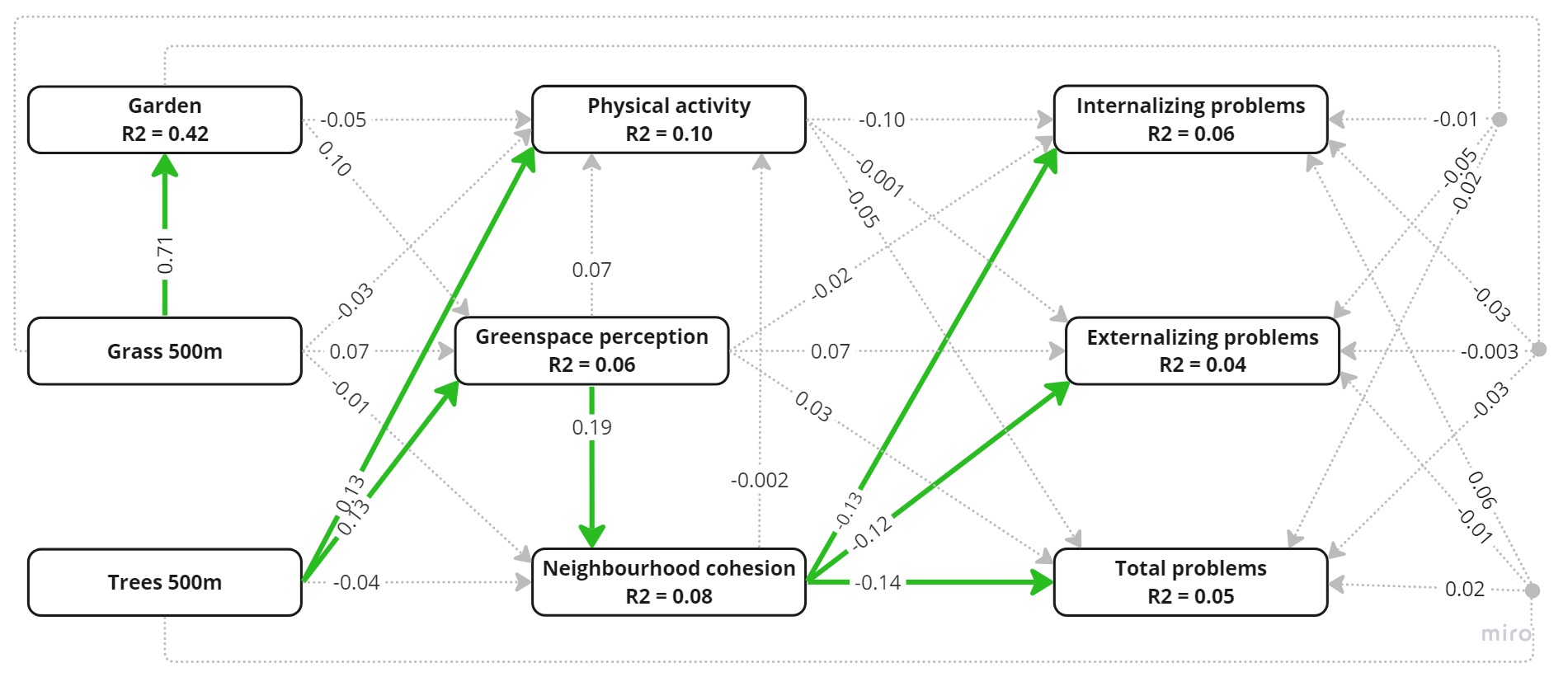


Figure S4: Diagram with path coefficients, estimated in **children without ADHD,** of the structural equation modelling (SEM) model for greenspace availability and behavioural problems with mediators physical activity, greenspace perception, and neighbourhood social cohesion and confounders age, sex, socio-economic status (SES), and town size. Coefficients for all but the dichotomous presence of garden are standardized. Green bold lines have path coefficients whose 95% confidence intervals do not contain zero. R2 shows the proportion of variance explained in each endogenous variable. Confounders, covariances and error terms are not displayed to enhance readability.

Table S5: Estimates and 95% bootstrap confidence intervals (CI) for direct, indirect, and total effects, estimated in **children without ADHD**, of the structural equation modelling (SEM) model for greenspace availability and behavioural problems with mediators physical activity, greenspace perception, and neighbourhood social cohesion and confounders age, sex, socio-economic status (SES), and town size. Effect estimates whose 95% CIs do not contain zero are shown in bold.

|  | Estimate (95% Confidence Interval) | | |
| --- | --- | --- | --- |
|  | Internalizing problems | Externalizing problems | Total problems |
| **Direct effects** |  |  |  |
| Grass 500m | -0.03 (-0.16 – 0.10) | -0.003 (-0.09 – 0.09) | -0.03 (-0.14 – 0.07) |
| Tree 500m | 0.06 (-0.02 – 0.15) | -0.01 (-0.07 – 0.07) | 0.02 (-0.05 – 0.10) |
| Garden | -0.01 (-0.13 – 0.13) | -0.05 (-0.16 – 0.04) | -0.02 (-0.13 – 0.09) |
| **Indirect effects via physical activity** |  |  |  |
| Grass 500m Physical activity | 0.003 (-0.01 – 0.02) | 0.000 (-0.01 – 0.01) | 0.002 (-0.01 – 0.01) |
| Tree 500m Physical activity | **-0.01 (-0.03 – -0.0003)** | 0.000 (-0.01 – 0.01) | -0.01 (-0.02 – 0.0004) |
| Garden Physical activity | 0.01 (-0.01 – 0.02) | 0.000 (-0.01 – 0.01) | 0.002 (-0.01 – 0.01) |
| **Indirect effects via greenspace perception** |  |  |  |
| Grass 500m Greenspace perception | -0.001 (-0.01 – 0.01) | 0.01 (-0.004 – 0.02) | 0.002 (-0.04 – 0.01) |
| Tree 500m Greenspace perception | -0.002 (-0.02 – 0.01) | 0.01 (-0.002 – 0.02) | 0.004 (-0.01 ­– 0.02) |
| Garden Greenspace perception | -0.002 (-0.02 – 0.01) | 0.01 (-0.003 – 0.02) | 0.003 (-0.01 – 0.02) |
| **Indirect effects via neighbourhood cohesion** |  |  |  |
| Grass 500m Neighbourhood cohesion | 0.002 (-0.01 – 0.02) | 0.002 (-0.01 – 0.02) | 0.002 (-0.01 – 0.02) |
| Tree 500m Neighbourhood cohesion | 0.01 (-0.01 – 0.02) | 0.01 (-0.01 – 0.02) | 0.01 (-0.01 – 0.02) |
| **Specific indirect effects** |  |  |  |
| Grass 500m Greenspace perception Physical activity | -0.001 (-0.002 – 0.001) | -0.000 (-0.001 – 0.001) | -0.0002 (-0.001 – 0.0004) |
| Trees 500m Greenspace perception Physical activity | -0.001 (-0.004 – 0.001) | -0.000 (-0.001 – 0.001) | -0.0004 (-0.002 – 0.001) |
| Garden Greenspace perception Physical activity | -0.001 (-0.003 – 0.001) | -0.000 (-0.001 – 0.001) | -0.0003 (-0.002 – 0.0004) |
| Grass 500m Neighbourhood cohesion Physical activity | -0.000 (-0.001 – 0.001) | -0.000 (-0.0002 – 0.0002) | -0.000 (-0.0003 – 0.0004) |
| Trees 500m Neighbourhood cohesion Physical activity | -0.000 (-0.001 – 0.001) | -0.000 (-0.0003 – 0.0003) | -0.000 (-0.0004 – 0.0004) |
| Grass 500m Greenspace perception Neighbourhood cohesion | -0.002 (-0.001 – 0.001) | -0.002 (-0.01 – 0.001) | -0.002 (-0.01 – 0.002) |
| Trees 500m Greenspace perception Neighbourhood cohesion | **-0.003 (-0.01 – -0.0004)** | **-0.003 (-0.01 – -0.001)** | **-0.003 (-0.01 – -0.001)** |
| Garden Greenspace perception Neighbourhood cohesion | -0.002 (-0.01 – 0.0002) | -0.002 (-0.01 – 0.0002) | **-0.003 (-0.01 – -0.0002)** |
| **Total indirect effects** |  |  |  |
| Grass 500m | 0.003 (-0.02 – 0.03) | 0.01 (-0.01 – 0.003) | 0.01 (-0.01 – 0.03) |
| Trees 500m | -0.01 (-0.04 – 0.02) | 0.01 (-0.01 – 0.04) | 0.003 (-0.02 – 0.03) |
| Garden | 0.002 (-0.02 – 0.02) | 0.01 (-0.01 – 0.02) | 0.01 (-0.01 – 0.02) |
| **Total** **effects** |  |  |  |
| Grass 500m | -0.02 (-0.16 – 0.11) | 0.003 (-0.08 – 0.10) | -0.03 (-0.13 – 0.08) |
| Trees 500m | 0.05 (-0.03 – 0.14) | 0.01 (-0.06 – 0.09) | 0.03 (-0.05 – 0.11) |
| Garden | -0.003 (-0.13 – 0.13) | -0.05 (-0.15 – 0.05) | -0.02 (-0.13 – 0.10) |

Table S6: Path coefficients and 95% bootstrap confidence intervals (CI), estimated in **children without ADHD**, of the structural equation modelling (SEM) model for greenspace availability and behavioural problems with mediators physical activity, greenspace perception, and neighbourhood social cohesion and confounders age, sex, socio-economic status (SES), and town size. Estimates whose 95% CIs do not contain zero are shown in bold.

| Estimate (95% Confidence Interval) | | | | | | | |
| --- | --- | --- | --- | --- | --- | --- | --- |
|  | Internalizing problems ~ | Externalizing problems ~ | Total problems ~ | Physical activity ~ | Greenspace perception ~ | Neighbourhood cohesion ~ | Garden ~ |
| Grass 500m | -0.03(-0.16 – 0.10) | -0.003 (-0.09 – 0.09) | -0.03 (-0.14 – 0.07) | -0.03 (-0.17 – 0.12) | 0.07 (-0.05 – 0.18) | -0.01 (-0.11 – 0.08) | **0.71 (0.56 – 0.90)** |
| Trees 500m | 0.06 (-0.02 – 0.15) | -0.01 (-0.07 – 0.07) | 0.02 (-0.05 – 0.10) | **0.13 (0.03 – 0.23)** | **0.13 (0.04 – 0.23)** | -0.04 (-0.15 – 0.06) |  |
| Garden | -0.01 (-0.13 – 0.13) | -0.05 (-0.16 – 0.04) | -0.02 (-0.13 – 0.09) | -0.05 (-0.19 – 0.09) | 0.10 (-0.01 – 0.22) |  |  |
| Greenspace perception | -0.02 (-0.12 – 0.09) | 0.07 (-0.01 – 0.15) | 0.03 (-0.06 – 0.13) | 0.07 (-0.04 – 0.17) |  | **0.19 (0.10 – 0.27)** |  |
| Neighbourhood cohesion | **-0.13 (-0.22 – -0.05)** | **-0.12 (-0.19 – -0.05)** | **-0.14 (-0.22 – -0.07)** | -0.002 (-0.10 – 0.10) |  |  |  |
| Physical activity | **-0.10 (-0.19 – -0.01)** | -0.0001 (-0.08 – 0.08) | -0.05 (-0.13 – 0.04) |  |  |  |  |
| Socio-economic status | -0.20 (-0.73 – 0.27) | -0.10 (-0.52 – 0.32) | -0.17 (-0.65 – 0.29) | **0.87 (0.36 – 1.53)** | 0.40 (-0.07 – 0.92) | **0.54 (0.08 – 1.08)** | **0.87 (0.18 – 1.68)** |
| Age | 0.05 (-0.04 – 0.14) | 0.02 (-0.05 – 0.09) | 0.02 (-0.05 – 0.10) | 0.07 (-0.04 – 0.16) | -0.02 (-0.11 – 0.07) |  |  |
| Sex | -0.11 (-0.28 – 0.05) | -0.05 (-0.19 – 0.08) | -0.02 (-0.18 – 0.12) | **0.25 (0.07 – 0.45)** | 0.10 (-0.07 – 0.29) |  |  |
| Town size | 0.09 (-0.11 – 0.29) | -0.07(-0.22 – 0.08) | 0.01 (-0.16 – 0.18) | -0.03(-0.24 – 0.18) | 0.05 (-0.15 – 0.27) | -0.30 (-0.50 – 0.12) | -0.51(-0.80 – 0.25) |


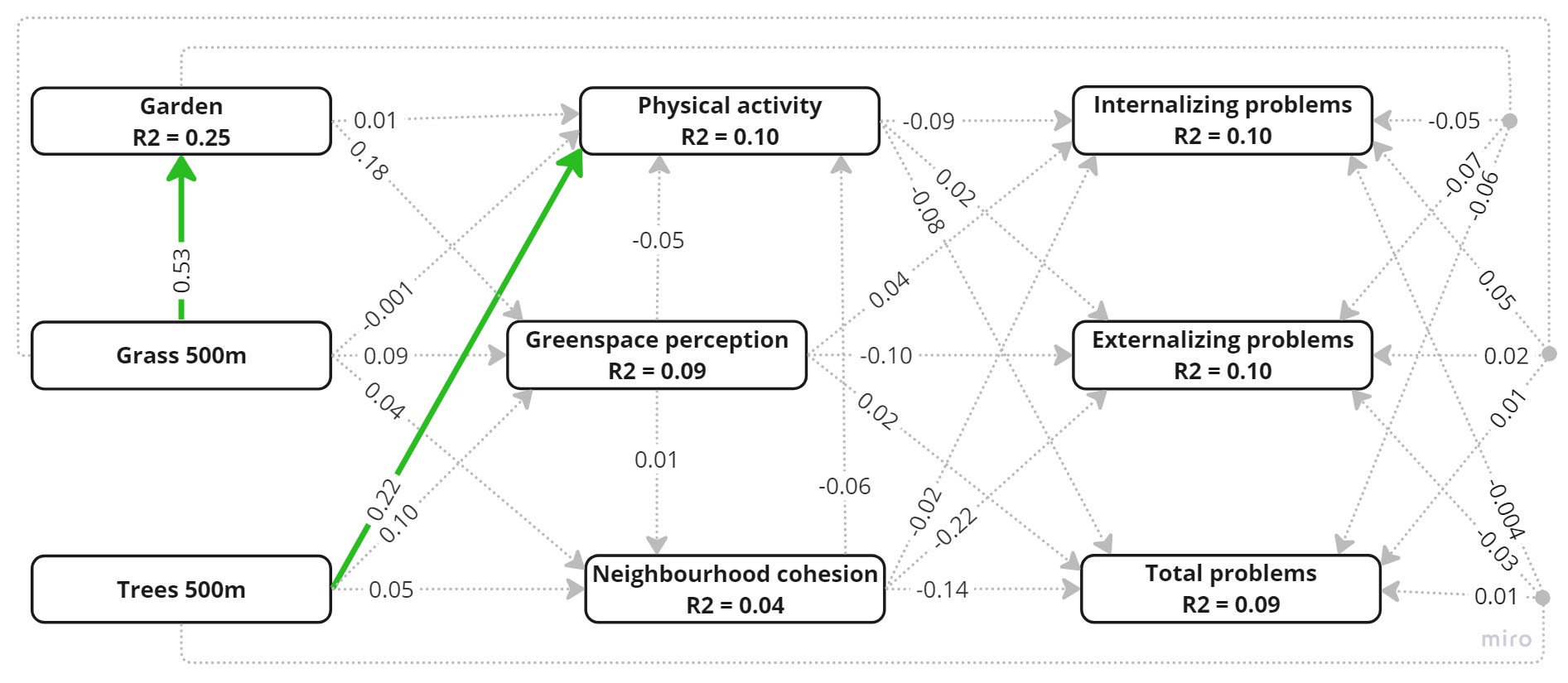


Figure S5: Diagram with path coefficients, estimated in **children with ADHD**, of the structural equation modelling (SEM) model for greenspace availability and behavioural problems with mediators physical activity, greenspace perception, and neighbourhood social cohesion and confounders age, sex, socio-economic status (SES), and town size. Coefficients for all but the dichotomous presence of garden are standardized. Green bold lines have path coefficients whose 95% confidence intervals do not contain zero. R2 shows the proportion of variance explained in each endogenous variable. Confounders, covariances and error terms are not displayed to enhance readability.

Table S7: Estimates and 95% bootstrap confidence intervals (CI) for direct, indirect, and total effects, estimated in **children with ADHD**, of the structural equation modelling (SEM) model for greenspace availability and behavioural problems with mediators physical activity, greenspace perception, and neighbourhood social cohesion and confounders age, sex, socio-economic status (SES), and town size. Effect estimates whose 95% CIs do not contain zero are shown in bold.

|  | Estimate (95% Confidence Interval) | | |
| --- | --- | --- | --- |
|  | Internalizing problems | Externalizing problems | Total problems |
| **Direct effects** |  |  |  |
| Grass 500m | 0.05 (-3.83 – 0.33) | -0.02 (-5.02 – 0.20) | 0.01 (-3.87 – 0.23) |
| Tree 500m | -0.004 (-3.64 – 0.20) | -0.03 (-4.61 – 0.17) | 0.01 (-3.25 – 0.21) |
| Garden | -0.05 (-0.36 – 4.17) | -0.07 (-0.46 – 4.47) | -0.06 (-0.44 – 3.39) |
| **Indirect effects via physical activity** |  |  |  |
| Grass 500m Physical activity | 0.000 (-0.11 – 3.17) | -0.000 (-0.05 – 4.33) | 0.000 (-0.09 – 3.19) |
| Tree 500m Physical activity | -0.02 (-0.12 – 3.56) | 0.004 (-0.06 – 4.06) | -0.02 (-0.10 – 2.92) |
| Garden Physical activity | -0.001 (-3.61 – 0.29) | 0.0002 (-3.98 – 0.42) | -0.001 (-3.32 – 0.32) |
| **Indirect effects via greenspace perception** |  |  |  |
| Grass 500m Greenspace perception | -0.004 (-0.08 – 1.00) | 0.01 (-0.09 – 1.01) | 0.002 (-0.10 – 0.94) |
| Tree 500m Greenspace perception | -0.004 (-0.23 – 0.31) | 0.01 (-0.23 – 0.54) | 0.002 (-0.27 – 0.43) |
| Garden Greenspace perception | -0.01 (-0.55 – 0.20) | 0.02 (-0.86 – 0.17) | 0.003 (-0.57 – 0.31) |
| **Indirect effects via neighbourhood cohesion** |  |  |  |
| Grass 500m Neighbourhood cohesion | -0.001 (-0.05 – 0.28) | -0.01 (-0.05 – 0.58) | -0.01 (-0.06 – 0.40) |
| Tree 500m Neighbourhood cohesion | -0.001 (-0.06 – 0.35) | -0.01 (-0.10 – 0.31) | -0.01 (-0.05 – 0.29) |
| **Specific** **indirect effects** |  |  |  |
| Grass 500m Greenspace perception Physical activity | 0.0004 (-0.80 – 0.05) | -0.0001 (-0.83 – 0.07) | 0.0004 (-0.78 – 0.08) |
| Trees 500m Greenspace perception Physical activity | 0.001 (-0.16 – 0.21) | -0.0001 (-0.42 – 0.19) | 0.0004 (-0.32 – 0.25) |
| Garden Greenspace perception Physical activity | 0.001 (-0.15 – 0.45) | -0.0002 (-0.17 – 0.62) | 0.001 (-0.23 – 0.29) |
| Grass 500m Neighbourhood cohesion Physical activity | 0.0002 (-0.24 – 0.02) | -0.000 (-0.48 – 0.02) | 0.0002 (-0.25 – 0.02) |
| Trees 500m Neighbourhood cohesion Physical activity | 0.0003 (-0.24 – 0.04) | -0.0001 (-0.23 – 0.09) | 0.0002 (-0.22 – 0.04) |
| Grass 500m Greenspace perception Neighbourhood cohesion | -0.000 ( -0.07 – 0.01) | -0.0003 (-0.07 – 0.02) | -0.0002 (-0.07 – 0.01) |
| Trees 500m Greenspace perception Neighbourhood cohesion | -0.000 (-0.05 – 0.01) | -0.0003 (-0.07 – 0.01) | -0.0002 (-0.07 – 0.01) |
| Garden Greenspace perception Neighbourhood cohesion | -0.000 (-0.02 – 0.02) | -0.001 (-0.05 – 0.03) | -0.0003 (-0.04 – 0.03) |
| **Total indirect effects** |  |  |  |
| Grass 500m | -0.004 (-0.13 – 3.89) | -0.0003 (-0.07 – 4.74) | -0.003 (-0.07 – 3.41) |
| Trees 500m | -0.02 (-0.15 – 4.08) | 0.003 (-0.08 – 4.66) | -0.02 (-0.11 – 3.22) |
| Garden | -0.01 (-3.62 – 0.34) | 0.02 (-4.07 – 0.49) | 0.003 (-3.33 – 0.52) |
| **Total effects** |  |  |  |
| Grass 500m | 0.04 (-0.22 – 0.23) | -0.02 (-0.29 – 0.15) | 0.01 (-0.22 – 0.18) |
| Trees 500m | -0.03 (-0.15 – 0.14) | -0.03 (-0.19 – 0.17) | -0.02 (-0.16 – 0.17) |
| Garden | -0.06 (-0.28 – 0.37) | -0.06 (-0.26 – 0.38) | -0.05 (-0.24 – 0.40) |

Table S8: Path coefficients and 95% bootstrap confidence intervals, estimated in **children with ADHD**, of the structural equation modelling (SEM) model for greenspace availability and behavioural problems with mediators physical activity, greenspace perception, and neighbourhood social cohesion and confounders age, sex, socio-economic status (SES), and town size. Estimates whose 95% CIs do not contain zero are shown in bold.

| Estimate (95% Confidence Interval) | | | | | | |  |
| --- | --- | --- | --- | --- | --- | --- | --- |
|  | Internalizing  problems ~ | Externalizing  problems ~ | Total  problems ~ | Physical  activity ~ | Greenspace  perception ~ | Neighbourhood cohesion ~ | Garden ~ |
| Grass 500m | 0.05 (-3.83 – 0.33) | -0.02 (-5.02 – 0.20) | 0.01 (-3.87 – 0.23) | -0.001 (-0.22 – 0.31) | 0.09 (-0.09 – 0.30) | 0.04 (-0.11 – 0.17) | **0.53 (0.35 – 0.76)** |
| Trees 500m | -0.004 (-3.64 – 0.20) | -0.03 (-4.61 – 0.17) | 0.01 (-3.25 – 0.21) | **0.22 (0.02 – 0.40)** | 0.10 (-0.06 – 0.23) | 0.05 (-0.11 – 0.19) |  |
| Garden | -0.05 (-0.36 – 4.17) | -0.07 (-0.46 – 4.47) | -0.06 (-0.44 – 3.39) | 0.01 (-0.43 – 0.25) | 0.18 (-0.06 – 0.38) |  |  |
| Greenspace perception | -0.04 (-1.57 – 4.63) | 0.10 (-1.72 – 3.94) | 0.02 (-1.23 – 4.98) | -0.05 (-0.44 – 0.35) |  | 0.01 (-0.21 – 0.19) |  |
| Neighbourhood cohesion | -0.02 (-0.51 – 6.12) | -0.22 (-0.84 – 8.94) | -0.14 (-0.80 – 5.71) | -0.06 (-0.41 – 0.16) |  |  |  |
| Physical activity | -0.09 (-0.42 – 16.25) | 0.02 (-0.24 – 20.04) | -0.08 (-0.33 – 13.98) |  |  |  |  |
| Socio-economic status | -0.99 (-197.85 – 1.69) | -0.72 (-359.31 – 0.96) | -0.79 (-210.55 – 1.35) | 0.79 (-1.73 – 15.11) | 0.48 (-2.94 – 5.90) | 0.79 (-1.36 – 7.79) | 0.78 (-1.14 – 6.50) |
| Age | 0.09 (-0.06 – 1.79) | 0.08 (-0.07 – 2.63) | 0.07 (-0.08 – 1.44) | -0.10 (-0.24 – 0.01) | -0.001 (-0.13 – 0.12) |  |  |
| Sex | -0.23 (-1.89 – 0.15) | 0.18 (-2.86 – 0.61) | 0.03 (-1.96 – 0.39) | 0.13 (-0.20 – 0.46) | -0.04 (-0.38 – 0.31) |  |  |
| Town size | 0.11 (-6.98 – 0.48) | 0.08 (-12.33 – 0.46) | 0.14 (-5.10 – 0.51) | 0.34 (-0.002 – 0.74) | -0.03 (-0.30 – 0.25) | -0.04 (-0.33 – 0.28) | 0.22 (-0.19 – 0.66) |


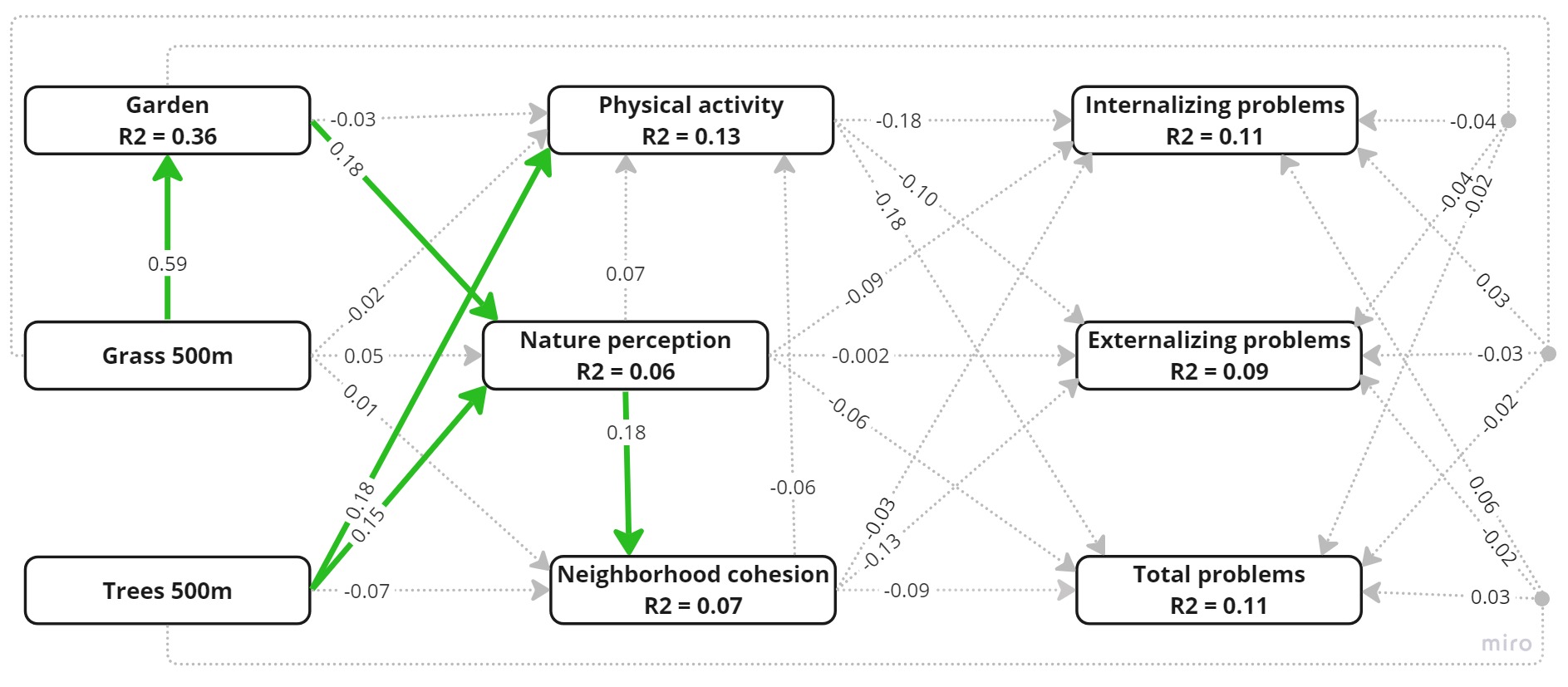


Figure S6: Diagram with path coefficients, estimated in the full sample, of the structural equation modelling (SEM) model for greenspace availability and behavioural problems with tree and grass cover computed for **males** around home addresses, mediators physical activity, greenspace perception, and neighbourhood social cohesion and confounders age, sex, socio-economic status (SES), and town size. Coefficients for all but the dichotomous presence of garden are standardized. Green bold lines have path coefficients whose 95% confidence intervals do not contain zero. R2 shows the proportion of variance explained in each endogenous variable. Confounders, covariances and error terms are not displayed to enhance readability.

Table S9: Estimates and 95% bootstrap confidence intervals (CI) for direct, indirect, and total effects, estimated in the full sample, of the structural equation modelling (SEM) model for greenspace availability and behavioural problems with tree and grass cover computed for **males** around home addresses, mediators physical activity, greenspace perception, and neighbourhood social cohesion and confounders age, sex, socio-economic status (SES), and town size. Effect estimates whose 95% CIs do not contain zero are shown in bold.

|  | Estimate (95% Confidence Interval) | | |
| --- | --- | --- | --- |
|  | Internalizing problems | Externalizing problems | Total problems |
| **Direct effects** |  |  |  |
| Grass 500m | 0.03(-0.11 – 0.17) | -0.02(-0.19 – 0.12) | -0.01(-0.16 – 0.13) |
| Tree 500m | 0.06(-0.04 – 0.15) | -0.02(-0.13 – 0.10) | 0.03(-0.09 – 0.14) |
| Garden | -0.03(-0.19 – 0.13) | -0.04(-0.21 – 0.15) | -0.02(-0.19 – 0.16) |
| **Indirect effects via physical activity** |  |  |  |
| Grass 500m Physical activity | 0.004(-0.02 – 0.04) | 0.002 (-0.02 – 0.03) | 0.004(-0.02 – 0.04) |
| Tree 500m Physical activity | -0.03(-0.06 – 0.005) | -0.02(-0.05 – 0.02) | -0.03(-0.07 – 0.007) |
| Garden Physical activity | 0.005(-0.03 – 0.04) | 0.003(-0.03 – 0.03) | 0.005(-0.03 – 0.04) |
| **Indirect effects via greenspace perception** |  |  |  |
| Grass 500m Greenspace perception | -0.004(-0.02 – 0.01) | -0.000(-0.009 – 0.01) | -0.003(-0.01 – 0.009) |
| Tree 500m Greenspace perception | -0.01(-0.04 – 0.001) | -0.0003(-0.02 – 0.02) | -0.009(-0.03 – 0.007) |
| Garden Greenspace perception | -0.02(-0.06 – 0.001) | -0.0003(-0.04 – 0.02) | -0.01(-0.05 – 0.009) |
| **Indirect effects via neighbourhood cohesion** |  |  |  |
| Grass 500m Neighbourhood cohesion | -0.0002(-0.005 – 0.008) | -0.0008(-0.02 – 0.02) | -0.0006(-0.01 – 0.01) |
| Tree 500m Neighbourhood cohesion | 0.002(-0.01 – 0.01) | 0.009(-0.005 – 0.03) | 0.007(-0.006 – 0.02) |
| **Specific** **indirect effects** |  |  |  |
| Grass 500m Greenspace perception Physical activity | -0.0006(-0.003 – 0.002) | -0.0003(-0.001 – 0.001) | -0.0006(-0.003 – 0.002) |
| Trees 500m Greenspace perception Physical activity | -0.002(-0.007 – 0.001) | -0.001(-0.005 – 0.002) | -0.002(-0.007 – 0.001) |
| Garden Greenspace perception Physical activity | -0.002(-0.01 – 0.002) | -0.001(-0.008 – 0.002) | -0.002(-0.01 – 0.002) |
| Grass 500m Neighbourhood cohesion Physical activity | 0.00006(-0.002 – 0.002) | 0.00003(-0.001 – 0.001) | 0.00006(-0.002 – 0.002) |
| Trees 500m Neighbourhood cohesion Physical activity | -0.0008(-0.006 – 0.002) | -0.0004(-0.004 – 0.002) | -0.0008(-0.006 – 0.002) |
| Grass 500m Greenspace perception Neighbourhood cohesion | -0.0003(-0.002 – 0.002) | -0.001(-0.004 – 0.003) | -0.0008(-0.003 – 0.002) |
| Trees 500m Greenspace perception Neighbourhood cohesion | -0.0009(-0.004 – 0.003) | -0.003(-0.009 – 0.0007) | -0.002( -0.008 – 0.002) |
| Garden Greenspace perception Neighbourhood cohesion | -0.001(-0.007 – 0.004) | -0.004(-0.014 – 0.0007) | -0.003(-0.01 – 0.002) |
| **Total indirect effects** |  |  |  |
| Grass 500m | -0.0006(-0.03 – 0.04) | 0.001(-0.02 – 0.03) | 0.0003(-0.03 – 0.04) |
| Trees 500m | **-0.04(-0.08 – -0.004)** | -0.01(-0.05 – 0.03) | -0.04(-0.08 – 0.009) |
| Garden | -0.01(-0.07 – 0.02) | 0.001(-0.05 – 0.03) | -0.008(-0.06 – 0.03) |
| **Total effects** |  |  |  |
| Grass 500m | 0.03(-0.11 – 0.17) | -0.02(-0.18 – 0.13) | -0.015(-0.16 – 0.13) |
| Trees 500m | 0.016(-0.07 – 0.10) | -0.03(-0.14 – 0.09) | -0.009(-0.11 – 0.11) |
| Garden | -0.05(-0.21 – 0.11) | -0.04(-0.20 – 0.14) | -0.03(-0.20 – 0.14) |

Table S10: Path coefficients and 95% bootstrap confidence intervals, estimated for **males**, of the structural equation modelling (SEM) model for greenspace availability and behavioural problems with mediators physical activity, greenspace perception, and neighbourhood social cohesion and confounders age, sex, socio-economic status (SES), and town size. Estimates whose 95% CIs do not contain zero are shown in bold.

| Estimate (95% Confidence Interval) | | | | | | |  |
| --- | --- | --- | --- | --- | --- | --- | --- |
|  | Internalizing  problems ~ | Externalizing  problems ~ | Total  problems ~ | Physical  activity ~ | Greenspace  perception ~ | Neighbourhood cohesion ~ | Garden ~ |
| Grass 500m | 0.03(-0.11 – 0.17) | -0.03(-0.19 – 0.13( | -0.02(-0.17 – 0.13) | -0.02(-0.18 – 0.16) | 0.05(-0.09 – 0.15) | 0.01(-0.10 – 0.10) | **0.59(0.46 – 0.76)** |
| Trees 500m | 0.06(-0.04 – 0.15) | -0.02(-0.13 – 0.10) | 0.03(-0.09 – 0.14) | **0.18(0.07 – 0.28)** | **0.15(0.05 – 0.24)** | -0.07(-0.20 – 0.03) |  |
| Garden | -0.04(-0.19 – 0.13) | -0.04(-0.21 – 0.15) | -0.02(-0.19 – 0.16) | -0.03(-0.24 – 0.15) | **0.18(0.06 – 0.34)** |  |  |
| Greenspace perception | -0.09(-0.22 – 0.01) | -0.002(-0.14 – 0.12) | -0.06(-0.20 – 0.05) | 0.07(-0.06 – 0.21) |  | **0.18(0.06 – 0.30)** |  |
| Neighbourhood cohesion | -0.03(-0.13 – 0.12) | -0.13(-0.25 – 0.04) | -0.09(-0.21 – 0.09) | -0.06(-0.31 – 0.07) |  |  |  |
| Physical activity | -0.18(-0.31 – 0.03) | -0.10(-0.26 – 0.10) | -0.18(-0.33 – 0.05) |  |  |  |  |
| Socio-economic status | -0.60(-3.19 – 0.23) | -0.69(-3.23 – 0.26) | -0.71(-3.29 – 0.16) | 1.36(0.33 – 4.07) | 0.11(-0.81 – 1.24) | 1.50(0.68 – 3.46) | 1.34(0.35 – 3.39) |
| Age | 0.07(-0.02 – 0.17) | 0.08(-0.03 – 0.18) | 0.06(-0.04 – 0.16) | -0.05(-0.15 – 0.06) | -0.01(-0.10 – 0.08) |  |  |
| Town size | 0.14(-0.06 – 0.34) | 0.03(-0.18 – 0.27) | 0.10(-0.12 – 0.32) | 0.07(-0.20 – 0.30) | 0.07(-0.12 – 0.29) | -0.17(-0.38 – 0.06) | -0.27(-0.56 – 0.002) |


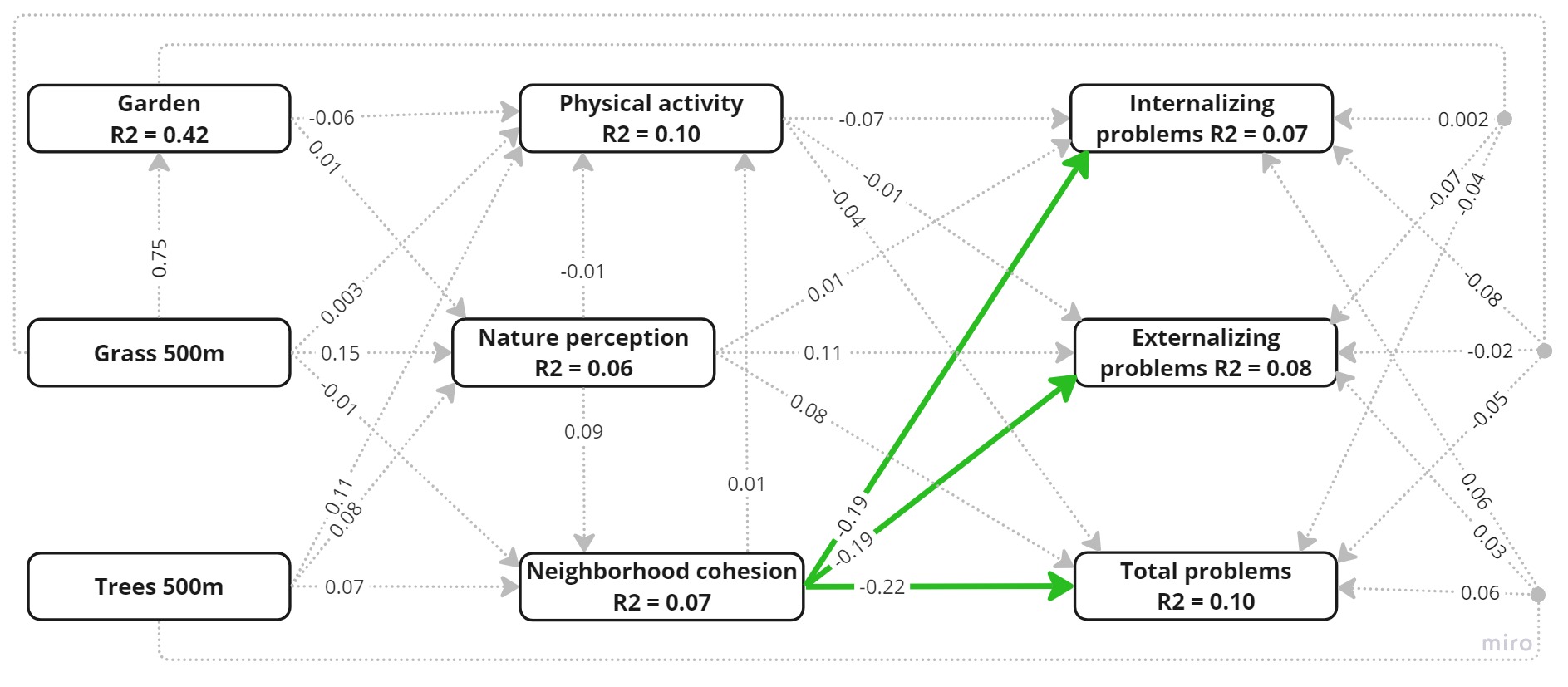


Figure S7: Diagram with path coefficients, estimated in the full sample, of the structural equation modelling (SEM) model for greenspace availability and behavioural problems with tree and grass cover computed for **female** around home addresses, mediators physical activity, greenspace perception, and neighbourhood social cohesion and confounders age, sex, socio-economic status (SES), and town size. Coefficients for all but the dichotomous presence of garden are standardized. Green bold lines have path coefficients whose 95% confidence intervals do not contain zero. R2 shows the proportion of variance explained in each endogenous variable. Confounders, covariances and error terms are not displayed to enhance readability.

Table S11: Estimates and 95% bootstrap confidence intervals (CI) for direct, indirect, and total effects, estimated in the full sample, of the structural equation modelling (SEM) model for greenspace availability and behavioural problems with tree and grass cover computed for **females** around home addresses, mediators physical activity, greenspace perception, and neighbourhood social cohesion and confounders age, sex, socio-economic status (SES), and town size. Effect estimates whose 95% CIs do not contain zero are shown in bold.

|  | Estimate (95% Confidence Interval) | | |
| --- | --- | --- | --- |
|  | Internalizing problems | Externalizing problems | Total problems |
| **Direct effects** |  |  |  |
| Grass 500m | -0.08(-0.30 – 0.16) | -0.02(-0.17 – 0.15) | -0.05(-0.24 – 0.16) |
| Tree 500m | 0.06(-0.05 – 0.21) | 0.03(-0.06 – 0.17) | 0.06(-0.05 – 0.19) |
| Garden | 0.002(-0.21 – 0.23) | -0.07(-0.24 – 0.09) | -0.04(-0.23 – 0.14) |
| **Indirect effects via physical activity** |  |  |  |
| Grass 500m Physical activity | -0.0003(-0.02 – 0.02) | -0.00002(-0.02 – 0.02) | -0.0001(-0.02 – 0.02) |
| Tree 500m Physical activity | -0.01(-0.03 – 0.01) | -0.001(-0.02 – 0.02) | -0.004(-0.03 – 0.01) |
| Garden Physical activity | 0.005(-0.01 – 0.03) | 0.0004(-0.02 – 0.02) | 0.003(-0.01 – 0.02) |
| **Indirect effects via greenspace perception** |  |  |  |
| Grass 500m Greenspace perception | 0.002(-0.02 – 0.03) | 0.02(-0.004 – 0.06) | 0.01(-0.01 – 0.05) |
| Tree 500m Greenspace perception | 0.001(-0.02 – 0.02) | 0.01(-0.01 – 0.03) | 0.01(-0.01 – 0.03) |
| Garden Greenspace perception | 0.0001(-0.02 – 0.01) | 0.001(-0.03 – 0.02) | 0.0005(-0.02 – 0.02) |
| **Indirect effects via neighbourhood cohesion** |  |  |  |
| Grass 500m Neighbourhood cohesion | 0.001(-0.02 – 0.03) | 0.001(-0.03 – 0.03) | 0.001(-0.03 – 0.03) |
| Tree 500m Neighbourhood cohesion | -0.01(-0.04 – 0.01) | -0.01(-0.04 – 0.01) | -0.02(-0.04 – 0.01) |
| **Specific** **indirect effects** |  |  |  |
| Grass 500m Greenspace perception Physical activity | 0.00007(-0.002 – 0.003) | 0.00001(-0.002 – 0.002) | 0.00004(-0.002 – 0.002) |
| Trees 500m Greenspace perception Physical activity | 0.00004(-0.001 – 0.002) | 0.000003(-0.001 – 0.001) | 0.00002(-0.001 – 0.001) |
| Garden Greenspace perception Physical activity | 0.000003(-0.001 – 0.001) | 0.0000002(-0.001 – 0.001) | 0.000001(-0.001 – 0.001) |
| Grass 500m Neighbourhood cohesion Physical activity | 0.00001(-0.001 – 0.001) | 0.0000005(-0.001 – 0.001) | 0.000003(-0.001 – 0.001) |
| Trees 500m Neighbourhood cohesion Physical activity | -0.00007(-0.002 – 0.002) | -0.00001(-0.001 – 0.001) | -0.00004(-0.001 – 0.001) |
| Grass 500m Greenspace perception Neighbourhood cohesion | -0.003( -0.01 – 0.001) | -0.002(-0.01 – 0.001) | -0.003(-0.01 – 0.001) |
| Trees 500m Greenspace perception Neighbourhood cohesion | -0.001(-0.01 – 0.001) | -0.001(-0.01 – 0.001) | -0.002(-0.01 – 0.001) |
| Garden Greenspace perception Neighbourhood cohesion | -0.00009(-0.004 – 0.004) | -0.00009(-0.004 – 0.004) | -0.0001(-0.004 – 0.005) |
| **Total indirect effects** |  |  |  |
| Grass 500m | 0.003(-0.04 – 0.05) | 0.02(-0.02 – 0.06) | 0.01(-0.03 – 0.06) |
| Trees 500m | -0.02(-0.06 – 0.01) | -0.01(-0.04 – 0.03) | -0.01(-0.06 – 0.02) |
| Garden | 0.005(-0.02 – 0.03) | 0.001(-0.03 – 0.03) | 0.003(-0.02 – 0.03) |
| **Total effects** |  |  |  |
| Grass 500m | -0.07(-0.29 – 0.16) | 0.002(-0.15 – 0.17) | -0.04(-0.22 – 0.16) |
| Trees 500m | 0.04(-0.07 – 0.18) | 0.03(-0.07 – 0.17) | 0.04(-0.07 – 0.18) |
| Garden | 0.01(-0.20 – 0.22) | -0.07(-0.23 – 0.09) | -0.04(-0.23 – 0.14) |

Table S12: Path coefficients and 95% bootstrap confidence intervals, estimated for **females**, of the structural equation modelling (SEM) model for greenspace availability and behavioural problems with mediators physical activity, greenspace perception, and neighbourhood social cohesion and confounders age, sex, socio-economic status (SES), and town size. Estimates whose 95% CIs do not contain zero are shown in bold.

| Estimate (95% Confidence Interval) | | | | | | |  |
| --- | --- | --- | --- | --- | --- | --- | --- |
|  | Internalizing  problems ~ | Externalizing  problems ~ | Total  problems ~ | Physical  activity ~ | Greenspace  perception ~ | Neighbourhood cohesion ~ | Garden ~ |
| Grass 500m | -0.08(-0.30 – 0.16) | -0.02(-0.17 – 0.15) | -0.05(-0.24 – 0.16) | 0.003(-0.19 – 0.20) | 0.15(-0.02 – 0.33) | -0.01(-0.13 – 0.13) | **0.75(0.55 – 1.01)** |
| Trees 500m | 0.06(-0.05 – 0.21) | 0.03(-0.06 – 0.17) | 0.06(-0.05 – 0.19) | 0.11(-0.02 – 0.25) | 0.08(-0.07 – 0.22) | 0.07(-0.04 – 0.19) |  |
| Garden | 0.002(-0.21 – 0.23) | -0.07(-0.24 – 0.09) | -0.04(-0.23 – 0.14) | -0.06(-0.25 – 0.12) | 0.01(-0.16 – 0.17) |  |  |
| Greenspace perception | 0.01(-0.13 – 0.17) | 0.11(-0.02 – 0.230 | 0.08(-0.05 – 0.22) | -0.01(-0.13 – 0.13) |  | 0.09(-0.02 – 0.19) |  |
| Neighbourhood cohesion | **-0.19(-0.33 – -0.06)** | **-0.19(-0.29 – -0.07)** | **-0.22(-0.33 – -0.10)** | 0.01(-0.15 – 0.15) |  |  |  |
| Physical activity | -0.07(-0.24 – 0.09) | -0.01(-0.15 – 0.14) | -0.04(-0.19 – 0.12) |  |  |  |  |
| Socio-economic status | -0.32(-1.14 – 0.14) | -0.21(-0.74 – 0.15) | -0.33(-0.94 – 0.10) | **0.59(0.11 – 1.10)** | 0.48(0.01 – 1.00) | -0.06(-0.39 – 0.37) | **0.60(0.05 – 1.21)** |
| Age | 0.01(-0.13 – 0.15) | -0.07(-0.19 – 0.04) | -0.06(-0.18 – 0.07) | 0.10(-0.02 – 0.23) | -0.03(-0.15 – 0.09) |  |  |
| Town size | 0.11(-0.15 – 0.42) | 0.02(-0.20 – 0.26) | 0.10(-0.14 – 0.38) | 0.06(-0.19 – 0.31) | -0.06(-0.31 – 0.22) | **-0.32(-0.56 – -0.10)** | -0.28(-0.66 – 0.06) |


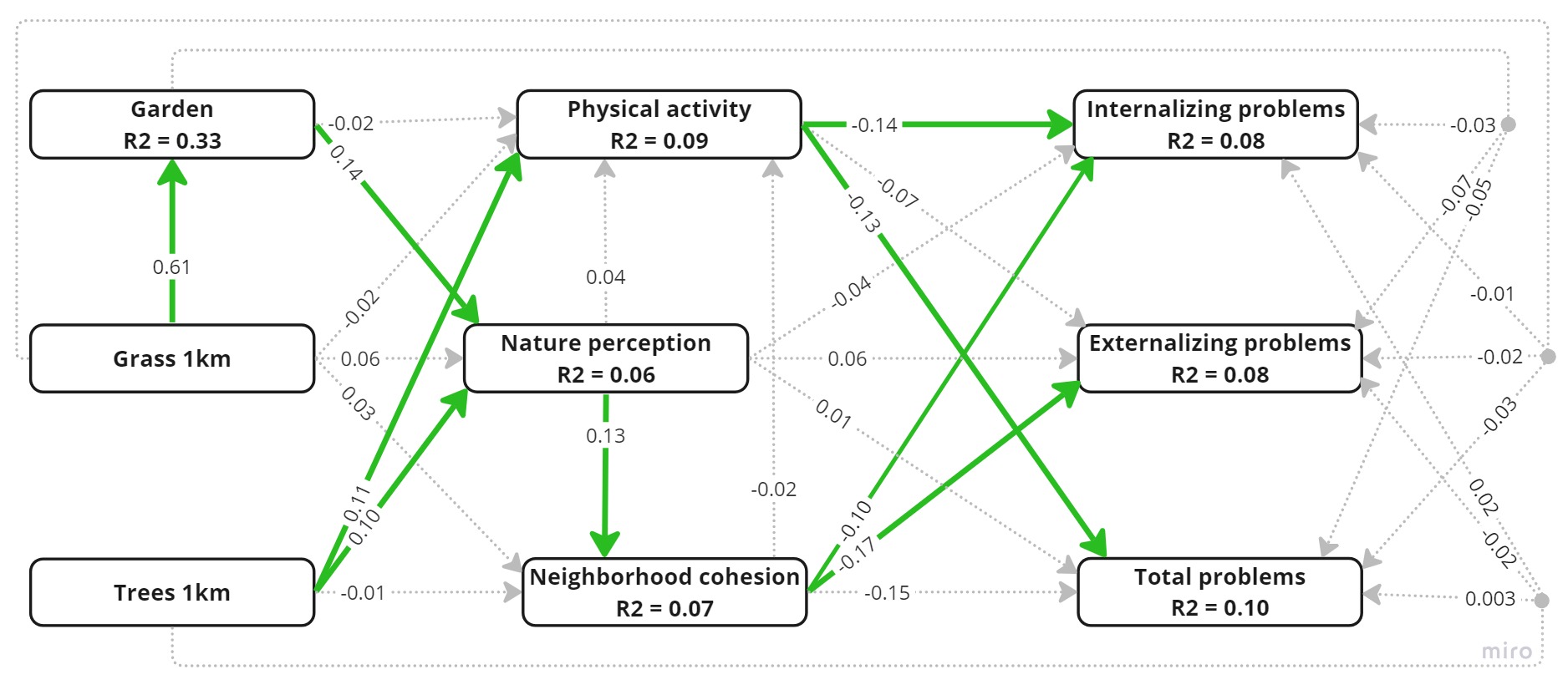


Figure S8: Diagram with path coefficients, estimated in the full sample, of the structural equation modelling (SEM) model for greenspace availability and behavioural problems with tree and grass cover computed in **1km buffers** around home addresses, mediators physical activity, greenspace perception, and neighbourhood social cohesion and confounders age, sex, socio-economic status (SES), and town size. Coefficients for all but the dichotomous presence of garden are standardized. Green bold lines have path coefficients whose 95% confidence intervals do not contain zero. R2 shows the proportion of variance explained in each endogenous variable. Confounders, covariances and error terms are not displayed to enhance readability.

Table S13: Estimates and 95% bootstrap confidence intervals (CI) for direct, indirect, and total effects, estimated in the full sample, of the structural equation modelling (SEM) model for greenspace availability and behavioural problems with tree and grass cover computed in **1km buffers** around home addresses, mediators physical activity, greenspace perception, and neighbourhood social cohesion and confounders age, sex, socio-economic status (SES), and town size. Effect estimates whose 95% CIs do not contain zero are shown in bold.

|  | Estimate (95% Confidence Interval) | | |
| --- | --- | --- | --- |
|  | Internalizing problems | Externalizing problems | Total problems |
| **Direct effects** |  |  |  |
| Grass 500m | -0.01 (-0.11 – 0.10) | -0.02 (-0.13 – 0.09) | -0.03 (-0.14 – 0.08) |
| Tree 500m | 0.02 (-0.05 – 0.10) | -0.02 (-0.10 – 0.06) | 0.003 (-0.07 – 0.09) |
| Garden | -0.03 (-0.15 – 0.08) | -0.07 (-0.18 – 0.04) | -0.05 (-0.17 – 0.06) |
| **Indirect effects via physical activity** |  |  |  |
| Grass 1km Physical activity | 0.002 (-0.01 – 0.02) | 0.001 (-0.01 – 0.01) | 0.002 (-0.01 – 0.02) |
| Tree 1km Physical activity | **-0.02 (-0.04 – -0.002)** | -0.01 (-0.03 – 0.002) | **-0.02 (-0.04 – -0.001)** |
| Garden Physical activity | 0.003 (-0.01 – 0.02) | 0.001 (-0.01 – 0.01) | 0.002 (-0.01 – 0.02) |
| **Indirect** **effects via greenspace perception** |  |  |  |
| Grass 1km Greenspace perception | -0.002 (-0.01 – 0.01) | 0.003 (-0.003 – 0.01) | 0.001 (-0.004 – 0.01) |
| Tree 1km Greenspace perception | -0.004 (-0.02 – 0.01) | 0.01 (-0.003 – 0.02) | 0.001 (-0.01 – 0.01) |
| Garden Greenspace perception | -0.01 (-0.02 – 0.01) | 0.01 (-0.01 – 0.02) | 0.001 (-0.01 – 0.01) |
| **Indirect effects via neighbourhood cohesion** |  |  |  |
| Grass 1km Neighbourhood cohesion | -0.003 (-0.01 – 0.01) | -0.01 (-0.02 – 0.01) | -0.01 (-0.02 – 0.01) |
| Tree 1km Neighbourhood cohesion | 0.001 (-0.01 – 0.01) | 0.002 (-0.01 – 0.02) | 0.002 (-0.01 – 0.02) |
| **Specific indirect** **effects** |  |  |  |
| Grass 1km Greenspace perception Physical activity | -0.0003 (-0.001 – 0.001) | -0.0002 (-0.001 – 0.0004) | -0.0003 (-0.001 – 0.001) |
| Trees 1km Greenspace perception Physical activity | -0.001 (-0.002 – 0.001) | -0.0003 (-0.001 – 0.0004) | -0.001 (-0.002 – 0.001) |
| Garden Greenspace perception Physical activity | -0.001 (-0.003 – 0.001) | -0.0004 (-0.002 – 0.001) | -0.001 (-0.003 – 0.001) |
| Grass 1km Neighbourhood cohesion Physical activity | 0.0001 (-0.001 – 0.001) | 0.0000 (-0.0004 – 0.001) | 0.0000 (-0.001 – 0.001) |
| Trees 1km Neighbourhood cohesion Physical activity | -0.0000 (-0.001 – 0.001) | -0.0000 (-0.001 – 0.0004) | -0.0000 (-0.001 – 0.001) |
| Grass 1km Greenspace perception Neighbourhood cohesion | -0.001 (-0.003 – 0.001) | -0.001 (-0.004 – 0.001) | -0.001 (-0.004 – 0.001) |
| Trees 1km Greenspace perception Neighbourhood cohesion | **-0.001 (-0.004 – -0.0002)** | **-0.002 (-0.01 – -0.001)** | **-0.002 (-0.01 – -0.0004)** |
| Garden Greenspace perception Neighbourhood cohesion | **-0.002 (-0.01 – -0.0002)** | **-0.003 (-0.01 – -0.001)** | **-0.003 (-0.01 – -0.001)** |
| **Total indirect effects** |  |  |  |
| Grass 1km | -0.004 (-0.02 – 0.02) | -0.001 (-0.02 – 0.02) | -0.003 (-0.02 – 0.02) |
| Trees 1km | -0.02 (-0.04 – 0.003) | -0.001 (-0.03 – 0.02) | -0.01 (-0.04 – 0.01) |
| Garden | -0.003 (-0.03 – 0.02) | 0.01 (-0.01 – 0.03) | 0.003 (-0.02 – 0.02) |
| **Total effects** |  |  |  |
| Grass 500m | -0.01 (-0.11 – 0.10) | -0.02 (-0.14 – 0.09) | -0.03 (-0.14 – 0.09) |
| Trees 500m | -0.002 (-0.07 – 0.08) | -0.02 (-0.10 – 0.06) | -0.01 (-0.09 – 0.07) |
| Garden | -0.04 (-0.15 – 0.08) | -0.06 (-0.17 – 0.04) | -0.05 (-0.16 – 0.06) |

Table 14: Path coefficients and 95% bootstrap confidence intervals (CI), estimated in the full sample, of the structural equation modelling (SEM) model for greenspace availability and behavioural problems with tree and grass cover computed in **1km buffers** around home addresses, mediators physical activity, greenspace perception, and neighbourhood social cohesion and confounders age, sex, socio-economic status (SES), and town size. Estimates whose 95% CIs do not contain zero are shown in bold.

| Estimate (95% Confidence Interval) | | | | | | | |
| --- | --- | --- | --- | --- | --- | --- | --- |
|  | Internalizing problems ~ | Externalizing problems ~ | Total  problems ~ | Physical  activity ~ | Greenspace  perception ~ | Neighbourhood cohesion ~ | Garden ~ |
| Grass 1km | -0.01 (-0.11 – 0.10) | -0.02 (-0.13 – 0.09) | -0.03 (-0.14 – 0.08) | -0.02 (-0.12 – 0.10) | 0.06 (-0.05 – 0.15) | 0.03 (-0.06 – 0.12) | **0.61 (0.49 – 0.76)** |
| Trees 1km | 0.02 (-0.05 – 0.10) | -0.02 (-0.10 – 0.06) | 0.003 (-0.07 – 0.09) | **0.11 (0.02 – 0.20)** | **0.10 (0.03 – 0.18)** | -0.01 (-0.10 – 0.07) |  |
| Garden | -0.03 (-0.15 – 0.08) | -0.07 (-0.18 – 0.04) | -0.05 (-0.17 – 0.06) | -0.02 (-0.13 – 0.09) | **0.14 (0.04 – 0.25)** |  |  |
| Greenspace perception | -0.04 (-0.12 – 0.05) | 0.06 (-0.03 – 0.13) | 0.01 (-0.07 – 0.09) | 0.04 (-0.04 – 0.13) |  | **0.13 (0.06 – 0.20)** |  |
| Neighbourhood cohesion | **-0.10 (-0.18 – -0.03)** | **-0.17 (-0.24 – -0.09)** | **-0.15 (-0.23 – -0.08)** | -0.02 (-0.10 – 0.07) |  |  |  |
| Physical activity | **-0.14 (-0.24 – -0.04)** | -0.07 (-0.17 – 0.02) | **-0.13 (-0.23 – -0.04)** |  |  |  |  |
| Socio-economic status | -0.40 (-0.97 – 0.06) | -0.36 (-0.86 – 0.07) | -0.43 (-1.00 – 0.02) | **0.87 (0.44 – 1.43)** | 0.37 (-0.04 – 0.85) | **0.56 (0.17 – 1.10)** | **0.74 (0.18 – 1.48)** |
| Age | 0.05 (-0.03 – 0.13) | 0.02 (-0.06 – 0.10) | 0.02 (-0.07 – 0.09) | 0.01 (-0.07 – 0.09) | -0.01 (-0.08 – 0.06) |  |  |
| Sex | 0.01 (-0.16 – 0.16) | **0.24 (0.09 – 0.39)** | **0.23 (0.09 – 0.37)** | 0.13 (-0.03 – 0.28) | 0.04 (-0.11 – 0.19) |  |  |
| Town size | 0.11 (-0.06 – 0.28) | 0.002 (-0.16 – 0.16) | 0.07 (-0.10 – 0.24) | 0.07 (-0.12 – 0.24) | 0.02 (-0.14 – 0.19) | **-0.22 (-0.38 – -0.07)** | **-0.24 (-0.46 – -0.02)** |

Table S15: Data extracted from existing studies on greenspace and behavioural problems.

|  | First author,  year (country) | Design | Population (n) | Green space type, buffer and source | Models’ adjustment | Outcome and assessment method | Results |
| --- | --- | --- | --- | --- | --- | --- | --- |
| 1 | Amoly et al., 2014 (Spain) | Cross-sectional | 7–10 years (2111) | NDVI - Satellite image (Landsat 5, 30 × 30 m resolution).  Major green spaces (not defined by type) 100, 250, 500 m.  Use (Time playing/spending) - Questionnaire. | Sex, school level, ethnicity, pre-term birth, breastfeeding, exposure to environmental tobacco smoke, maternal smoking during pregnancy, responding person, parental educational achievement, parental employment status, parental marital status, and Neighbourhood socioeconomic status based on quartiles of 2010 household income. | Total difficulties; hyperactivity/inattention and ADHD symptoms; Emotional symptoms; Conduct problems; Peer problems; Prosocial behavior  DSM-IV; SDQ, Rated by parents | Found statistically significant inverse associations between  Greenspace playing time -> total difficulties, emotional symptoms, peer relationship problems  Residential surrounding greenness -> total difficulties, hyperactivity/inattention and ADHD/DSM-IV total and inattention scores  Annual beach attendance -> total difficulties, peer relationship problems, and prosocial behavior. |
| 2 | Andrusaityte, et al., 2020 (Lithuania) | Cross-sectional | 4–6 years (1489) | NDVI -Satellite (Unknown).  Use (Time spent in a city park)  City parks, Agricultural green places, or nature conservation areas, and part of city forests) 100, 300, 500 m. | Sex, birth order, breastfeeding, antibiotic usage during the first postnatal year, wheeze during 12 months, clinically diagnosed asthma, allergy, underweight, tobacco smoke, the parents' socio-economic status, birth weight, PM2.5, and NO2. | Total difficulties; Hyperactivity/inattention; Emotional symptoms; Conduct problems; Peer problems; Prosocial behavior.  Lithuanian version of the Strengths & Difficulties Questionnaire (SDQ) for parents | Increase in sedentary behaviour (the time spent at the computer and the TV watching time (>21h/week)) → child with poor health.  +1h/week in parks → decreased sedentary behaviour, prosocial behaviour & lower risk of poor health  Lower residential greenness -> poorer mental and general health. |
| 3 | Balseviciene et al., 2014  (Lithuania) | Cross-sectional | 4–6 years  (1468) | NDVI; Satellite image (Landsat 7, 30 × 30 m resolution).  City parks, agricultural green places, or nature conservation areas, and part of city forests) 300m.    Spatial land cover map (Kaunas municipality, parks >1 ha). | No | Total difficulties; Hyperactivity/inattention  ; Emotional symptoms;  Conditional problems; Peer problems;  Prosocial behavior.  Lithuanian version of the Strengths & Difficulties Questionnaire (SDQ) for parents | Farther residential distance from parks -> worse mental health (except for the emotional problems subscale) in children whose mothers had a lower education level.  More residential greenness -> worse mental health (more conditional problems and less prosocial behavior) in children whose mothers had a higher education level. |
| 4 | Bijnens et al., 2020 (Belgium) | Cross-sectional | 7–15 years (620) | Semi-natural, forested, and urban green areas 50, 100, 300, 500, 1000, 2000m around the prenatal and childhood residential address.  Corine land cover, and Green Map of Flanders (1m2 resolution). | Sex, age, parental education, Neighbourhood household income, year of assessment and zygosity and chorionicity. | Total difficulties; Externalize difficulties; Internalized difficulties.  CBCL | Higher residential greenspace -> higher intelligence & lower behavioural problems children living in urban areas.  Level of urbanicity modified the association of greenspace exposure with intelligence and behavior in childhood |
| 5 | Dockx et al 2021 (Belgium) (ENVIRONAGE birth cohort) | Follow-up | 4-6 years  (411) | Green spaces were calculated based on high-resolution land cover data (1 x 1m) within 50m, 100m, 300m, and 500m and 1000m around the residence.    Green map of Flanders. | Covariables: Age and sex of child, maternal education | Strengths and Difficulties Questionnaire (SDQ) | Residential surrounding green space -> reduction in hyperactive behavioral traits, beneficial effect on motor accuracy and improved visual memory-related tasks |
| 6 | Dzhambov et al. 2022 (Austria and Italy) | Cross-sectional | 8-12 years (1251) | Estimated nature exposure using a comprehensive naturalness index called “distance to nature” (D2N).  This was calculated in 100, 500, and 1000- m radii around the child’s home and 100m around school.  Presence of a home garden was assessed by self-report. | Potential mediators: parental smoking in the home, sleep problems, body mass index, and Systemic inflammation (urinary neopterin concentrations and cotinine concentrations).    Potential confounders: child’s sex and age, maternal education, house type, and geographic region. | Needleman questionnaire for classroom performance rated by teachers. (3 broad domains : distractibility, hyperactivity and performance). | Home with a garden and school closer to nature -> less school behavior problems.  Associations were in unexpected directions between behavior problems and residential proximity to nature and depended on the outcome and context. |
| 7 | Feng et al., 2017a (Australia) | Cross-sectional | 12–13 years (3083) | Parks  Land use data (Australian Bureau of Statistics, parkland, mesh block level),  Quality - Quality (by questionnaire) | Indicators of area disadvantage and geographic remoteness, maternal education, child age and gender. | Total difficulties; Externalize difficulties; Internalized difficulties  SDQ | Higher green space quantity and quality -> favorable child mental wellbeing  Green space quantity -> total difficulties score and the internalising subscale. Green space quality were consistently observed for both parent- and child-reported outcomes.  Teacher-reported outcomes were not significantly associated with green space exposure. |
| 8 | Feng et al., 2017b  (Australia) | Longitudinal | 4–5 years  (4968) | Parks    Percent coverage | Demographic and socioeconomic confounders. | Total difficulties; Externalize difficulties;  Internalized difficulties    SDQ | Children living in areas with 21-40% greenspace -> experienced most favorable mental wellbeing.  Green space quantity and quality count for well-being in childhood and, as children grow older, the well-being relevance of having access to higher quality green space becomes more salient. |
| 9 | Jimenez et al., 2021  (USA) | Longitudinal | Birth −7 years  (908) | Surrounding greenness  90, 270, 1230 m    NDVI around participant’s residential addresses at a 90 m, buffer size. NDVI was calculated m and 1230m from Landsat satellite data at 30 m resolution.    NDVI - Satellite images (Landsat, 30 × 30 resolution) | Time invariant covariates: mother’s characteristics: age at enrollment, maternal IQ, smoking during pregnancy, college education, marital status  Child’s characteristics: age at the time of assessment, sex, race/ethnicity, season of birth    Time-varying covariates: household income, median census tract income, population density. | Total difficulties (composed of peer, hyperactivity, emotional, and conduct SDQ sub-scales).  ; Prosocial behavior;  Externalize difficulties; Internalized  Difficulties      SDQ | “persistent” exposure to maximum greenness at birth, early childhood and mid-childhood (vs. minimum) -> behavior mid-childhood.  Early childhood onset (vs minimum at all time points) -> protective association with the total difficulties at mid-childhood  No associations were observed with early adolescent outcomes. |
| 10 | Liao et al., 2020 (China) | Cross-sectional | 5–6 years (6039) | Surrounding greenness  100 m    NDVI calculated using Landsat 5 Thematic Mapper (TM) and Landsat 8 TM data, with a high spatial resolution of30 x 30m in 100m buffer around residence and Kindergarden.    NDVI - Satellite images (Landsat 5, 30 × 30 resolution) | Covariates: residential areas, maternal age and education, parity, preterm birth, newborn’s weight-for-length Z scores, breastfeeding during the first month after delivery, children’s age and sex. | Total difficulties; Internalizing; Externalizing problems        CBCL | 1 IQR increase in kindergarten and residence–kindergarten-weighted NDVI -> decreased total behaviour problem, anxiety, depression, aggression, hyperactivity, attention deficit behaviour |
| 11 | Madzia et al., 2019  (USA) | Longitudinal | 7 and 12 years  (562) | Surrounding greenness  200, 400, 800 m    NDVI - Satellite images (Landsat, 100 × 100 resolution) | Neighbourhood deprivation, maternal education, race, and sex | externalizing behaviors including hyperactivity, attention problems, aggression, and conduct problems, and internalizing behaviors, including depression, anxiety, and somatization,  BASC-2 | At age 7 years, a 0.1-unit increase in NDVI -> decreased conduct scores.  At age 12 years, a 0.1-unit increase in NDVI -> decrease in anxiety scores, decreased depression scores, and decreased somatization scores. |
| 12 | Markevych et al., 2014  (Germany) | Cross-sectional | 10 years  (1932) | Cemetery, garden, park, plant nursery 500 m    NDVI - Satellite images (Landsat5, 30 × 30 resolution)    Proximity - Bavarian land-use dataset (resolution <5 m) | study, sex, age, parental level of education, age of mother at time of birth, single parent status at the 10-year follow-up, time spent in front of a screen and time spent  outdoors. | Total difficulties; Hyperactivity/inattention; Emotional symptoms; Conduct problems; Peer  Problems      SDQ | Poor access to urban green spaces was -> more behavioural problems, consistent results for hyperactivity/inattention problems. |
| 13 | McEachan et al., 2018  (UK) | Cross-sectional | 4 years  (2594) | Major green spaces (not clear type) 100, 300, 500 m    NDVI; Satellite images (Landsat 5, 30 × 30 resolution);  Proximity; For proximity the data source was unclear;  Satisfaction; Use; Availability Questionnaire  Percent coverage Survey  Putra et al. 2021 Quality Parent rating on Likert scale | Model 2: adjusted for child age, child sex, maternal age, and cohabitation status demographics plus maternal education, subjective poverty, household size, and Index of Multiple Deprivation (IMD).  Controlled further for deprivation plus maternal smoking, and mother’s treatment for common mental disorder in previous year. | Total difficulties; Externalize difficulties;  Internalized difficulties; Prosocial behavior      SDQ | More green space was -> with fewer internalising behavioural difficulties and with fewer total behavioural difficulties only for south Asian children.  Satisfaction with green space -> fewer total, internalising behavioural difficulties and greater prosocial behaviour (Among south Asian children)  No such associations were observed among white British children.  Positive effects of green space on wellbeing differ by ethnicity |
| 14 | Putra et al. 2021 (Australia) | Longitudinal | 4-5 years,  14-15 years  (4969) | Availability of good parks, playgrounds, and play space    Green space quality (parks, playground and play spaces in Neighbourhood ) | Greenspace use, pro-social behaviour and influence the green space-prosocial behaviour association.  Covariates: Child’s age, sex, ethnicity indicators including if the child was indigenous and if the child spoke a language other than the English at home, family socio-economic status, such as educational level of the parent who had the highest qualification in the family. | Prosocial behaviour that covers positive behaviours (e.g. sharing, helping) was measured using a prosocial scale from Goodman’s Strengths and Difficulties Questionnaire (SDQ)        . | Green space quality -> child prosocial behaviour.  Boys and younger children tended to benefit more from quality green space. |
| 15 | Richardson et al., 2017  (Scotland) | Longitudinal | 4–6 years  (2650) | Area of public parks and total natural space around each child's home (500m buffer), using 2011 data obtained ‘Scotland's Greenspace Map.    Percent coverage, Land use map (Corine land cover) | (only in sensitivity analysis)****Covariates: age, age2, sex, screen time, household educational attainment, household equivalized income, carer's mental health, and neighbourhood deprivation. | Total difficulties; Hyperactivity/inattention;  Emotional symptoms; Conditional problems;  Peer problems; Prosocial behavior.    SDQ  Social, emotional and behavioural difficulty scores by Strengths and Difficulties Questionnaire (SDQ). | Private garden access -> most SDQ domains.  More park or total natural space around homes -> better social, emotional and behavioural outcomes, |
| 16 | Ward et al. 2016 | Observational study | 11-14 years (108) | Locational data were gathered using the Qstarz BT-Q1000XT GPS receiver | Moderate-to-vigorous physical activity (MVPA) and green space exposure | Emotional wellbeing: The Life Satisfaction Scale (LSS), Ten Domain Index of Wellbeing (TDIW),  measure of happiness with life as a whole (HS).  Sensation seeking: Short form sensation seeking scale (SFSSS)  Risk-taking: youth version of the Balloon Analogue Risk Task (BART-Y)  CNS Vital Signs (CNS-VS): visual memory, verbal memory, processing speed, psychomotor speed, reaction time, cognitive flexibility, and executive function. | Greenspace exposure -> MVPA  Greenspace exposure and MVPA -> greater emotional wellbeing, with the former exhibiting a stronger relationship than the latter.  Risk-taking and sensation seeking scores -> MVPA. |
| 17 | Younan et al. 2016 (USA) | Longitudinal | 9-18 years  1287 | Neighbourhood greenspace was abstracted as the average of NDVI in buffers of 250-, 350-, 500-, and 1000-meters around each residence. | Confounders: age, gender, ethnicity, household SES, self-perceived Neighbourhood quality, and ambient temperature. | Aggressive behavior      CBCL | Both short-term (1- to 6-months) and long-term (1- to 3-years) exposures to  greenspace within 1,000-meters surrounding residences -> with reduced aggressive behaviors. |
